# BMP‐2 Signaling and Mechanotransduction Synergize to Drive Osteogenic Differentiation via YAP/TAZ

**DOI:** 10.1002/advs.201902931

**Published:** 2020-06-16

**Authors:** Qiang Wei, Andrew Holle, Jie Li, Francesca Posa, Francesca Biagioni, Ottavio Croci, Amelie S. Benk, Jennifer Young, Fatima Noureddine, Jie Deng, Man Zhang, Gareth J. Inman, Joachim P. Spatz, Stefano Campaner, Elisabetta A. Cavalcanti‐Adam

**Affiliations:** ^1^ College of Polymer Science and Engineering State Key Laboratory of Polymer Materials and Engineering Sichuan University Chengdu 610065 China; ^2^ Department of Cellular Biophysics Max Planck Institute for Medical Research Jahnstraße 29 Heidelberg 69120 Germany; ^3^ Department of Biophysical Chemistry Heidelberg University INF 253 Heidelberg 69120 Germany; ^4^ Department of Clinical and Experimental Medicine Medical School University of Foggia Foggia 71122 Italy; ^5^ Center for Genomic Science of IIT@SEMM Istituto Italiano di Tecnologia (IIT) Via Adamello 16 Milan 20139 Italy; ^6^ Growth Factor Signalling and Squamous Cancers Cancer Research UK Beatson Institute Garscube Estate Glasgow G61 1BD UK; ^7^ Institute of Cancer Sciences University of Glasgow Glasgow G12 8QQ UK; ^8^ Central Scientific Facility “Cellular Biotechnology,” MPI for Medical Research Jahnstr. 29 Heidelberg 69120 Germany

**Keywords:** BMP‐2, cell differentiation, mechanotransduction, signaling, YAP/TAZ

## Abstract

Growth factors and mechanical cues synergistically affect cellular functions, triggering a variety of signaling pathways. The molecular levels of such cooperative interactions are not fully understood. Due to its role in osteogenesis, the growth factor bone morphogenetic protein 2 (BMP‐2) is of tremendous interest for bone regenerative medicine, osteoporosis therapeutics, and beyond. Here, contribution of BMP‐2 signaling and extracellular mechanical cues to the osteogenic commitment of C2C12 cells is investigated. It is revealed that these two distinct pathways are integrated at the transcriptional level to provide multifactorial control of cell differentiation. The activation of osteogenic genes requires the cooperation of BMP‐2 pathway‐associated Smad1/5/8 heteromeric complexes and mechanosensitive YAP/TAZ translocation. It is further demonstrated that the Smad complexes remain bound onto and active on target genes, even after BMP‐2 removal, suggesting that they act as a “molecular memory unit.” Thus, synergistic stimulation with BMP‐2 and mechanical cues drives osteogenic differentiation in a programmable fashion.

## Graphical abstract

Bone morphogenetic protein 2 (BMP‐2) signaling and mechanotransduction pathways are integrated at the transcriptional level to provide multifactorial control of cell differentiation. The activation of osteogenic genes requires the cooperation of BMP‐2 pathway‐associated Smad1/5/8 heteromeric complexes and mechanosensitive YAP/TAZ translocation. The Smad complexes remain bound onto and active on target genes, even after BMP‐2 removal, suggesting that they act as a “molecular memory unit.”

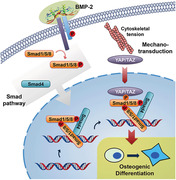

## 1 Introduction

Chemical and physical cues act upon cells to mediate a wide range of behaviors, including growth, differentiation, and survival.^[^ ^1^, ^2^ ^]^ Mechanotransduction enables cells to sense and respond to physical cues like extracellular matrix (ECM) viscoelasticity, ligand density, and topography.^[^ ^3^, ^4^, ^5^, ^6^ ^]^ These mechanical cues are translated into biochemical signals, ultimately activating nuclear transcription factors that control gene transcription for downstream molecular outputs and phenotypic maintenance or diversity.^[^ ^7^, ^8^ ^]^ Separately, biochemical cues provided by soluble growth factors regulate a variety of cellular processes, including the induction of cell fate during development. Growth factors initiate numerous signaling cascades by binding to and activating complementary receptors.^[^ ^9^ ^]^ While the activity of several growth factors has been shown to be dependent on mechanical cues,^[^ ^10^ ^]^ the crosstalk between mechanotransduction pathways and growth factor signaling is not yet well‐understood.

Bone morphogenetic protein 2 (BMP‐2) belongs to the transforming growth factor β (TGF‐β) superfamily. Beyond its canonical role in initiating the differentiation of osteoprogenitor cells into mature osteoblasts, it also is capable of stimulating the transdifferentiation of non‐osteogenic cells into osteoblasts.^[^ ^11^ ^]^ The BMP‐2 signaling cascade is activated via interactions between BMP‐2 and its heteromeric transmembrane receptor composed of types I and II serine/threonine kinase receptors. This activation induces recruitment and phosphorylation of Smad family signal transducing proteins Smad1/5/8. Heteromeric complexes are subsequently formed between phosphorylated Smad1/5/8 and the common mediator Smad4. These complexes then accumulate in the nucleus^[^ ^12^ ^]^ to regulate target gene transcription.^[^ ^13^, ^14^ ^]^

Several previous studies have noted that the efficiency of BMP‐2‐induced osteogenic differentiation is highly dependent on cell shape, cytoskeletal tension, cell–ligand interactions, and matrix stiffness.^[^ ^15^, ^16^, ^17^, ^18^ ^]^ In other words, BMP‐2 signaling and mechanotransduction pathways appear tightly interconnected.^[^ ^14^, ^19^ ^]^ A biological example of crosstalk related to cell shape regulation exists in the soft articular joints or soft bone marrow, where chondrogenic progenitor or adipogenic precursor cells, respectively, exhibit a spherical phenotype and resist being forced into an osteogenic fate when exposed to local BMP gradients.^[^ ^15^ ^]^

Here, we examine how mechanical cues, that is, matrix stiffness and cytoskeletal tension, affect biochemical signaling in a key step of the BMP‐2‐induced Smad1/5/8 signaling cascade. We then identify which step of osteogenesis towards an osteogenesis lineage is suppressed when cells lack cytoskeletal tension. Hippo pathway effectors YAP (Yes‐associated protein) and TAZ (transcriptional coactivator with PDZ‐binding motif, also known as WWTR1), which are well‐known mechanosensitive mediators of mechanical cues,^[^ ^8^ ^]^ are highlighted as key transcriptional regulators that coactivate osteogenic genes with Smad1/4/5/8 heteromeric complexes. Finally, we provide evidence that Smad signaling activity persists even after the removal of BMP‐2 in a process that serves as a “molecular memory unit” for BMP‐2 signaling. Ultimately, programmable BMP‐2 stimulation and mechanical cues were synergistically utilized to induce and enhance osteogenic differentiation.

## 2 Results

### 2.1 Cytoskeletal Tension Mediates BMP‐2‐Induced Osteogenic Differentiation

To investigate the role of mechanotransduction in BMP‐2 signaling, we monitored BMP‐2‐induced osteogenic differentiation of C2C12 mouse myoblasts on polyethylene glycol (PEG) hydrogels, which were functionalized with RGD peptides to promote cell adhesion. C2C12 cells are myoblasts that are able to transdifferentiate into osteoblasts upon stimulation with BMPs and represent an established in vitro model system to study BMP‐2 signaling.^[^ ^20^, ^21^ ^]^ C2C12s are a compelling cell line for untangling the independent contributions of both substrate stiffness and BMP‐2 signaling pathways precisely because their osteogenic differentiation is dependent upon both, rather than just one (as is the case with mesenchymal stem cells (MSCs)).^[^ ^22^ ^]^ In C2C12s, osteogenic differentiation is only observed when cells are treated with BMP‐2 and are exposed to high levels of substrate stiffness. By altering these two variables independently, we can better understand the dynamic role each modality plays in the onset of osteogenesis. In MSCs, the activation of osteogenetic genes can be provoked by either BMP‐2 signaling or substrate stiffness. Thus, perturbing one pathway will not result in a relative loss of osteogenesis, preventing us from understanding the interplay between the two modalities. Therefore, the C2C12 model is a powerful tool for investigating the synergistic role of matrix mechanics and BMP‐2 signaling in osteogenic differentiation.

**Figure 1:**
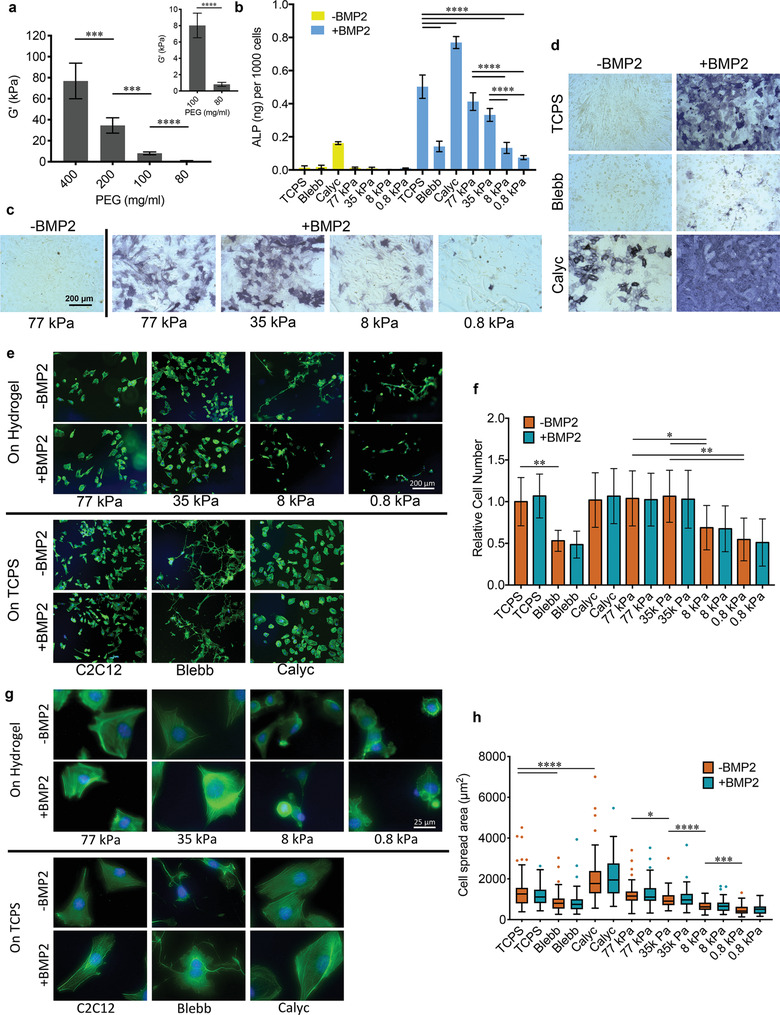
BMP‐2‐induced osteogenic differentiation of C2C12 cells. a) Average elastic modulus of PEG hydrogels (error bars are SD, *n* = 3, two technical replicates, Welch's *t*‐test). b) Quantitative assay of ALP activity in C2C12 cells cultured in different conditions for 7 days (*n* = 2–3, two technical replicates, one‐way ANOVA followed by post hoc Tukey's multiple comparisons test). c) Representative images of ALP staining for C2C12 cells on PEG hydrogels of different stiffness for 5 days. d) Representative images of ALP staining for C2C12 cells on TCPS with or without BMP‐2 and inhibitor treatment for 7 days. e) Representative images of F‐actin staining of C2C12 cells cultured in different conditions for 24 h. f) The number of attached C2C12 cells related to cells on TCPS without BMP‐2 treatment (*n* = 5, two technical replicates, Welch's *t*‐test). g) Representative high‐magnification images of F‐actin staining of C2C12 cells cultured in different conditions for 24 h. h) Spread area of C2C12 cells cultured in different conditions for 24 h (*n* = 50, two technical replicates, Welch's *t*‐test). TCPS: cells were cultured on TCPS; Blebb: cells were treated with blebbistatin; Calyc: cells were treated with calyculin A; X kPa: cells were cultured on hydrogels with stiffness X kPa.

Hydrogel stiffness was tuned by varying the concentration of PEG diacrylate, modulating the number of backbone polymers within the hydrogel network. The elastic moduli of the hydrogels, measured by rheometry, ranged from 0.8 to 77 kPa (**Figure** **1**a), within the range of physiological elasticity of natural tissues.^[^ ^22^ ^]^ PEG hydrogels prevent the nonspecific adsorption of proteins,^[^ ^23^ ^]^ allowing cells to directly sense matrix stiffness via integrin–RGD ligand interactions.^[^ ^24^, ^25^ ^]^

Osteogenic differentiation of C2C12 cells on the hydrogels was evaluated by quantifying alkaline phosphatase (ALP) activity, as well as staining for ALP expression (Figure 1b–d). Following addition of BMP‐2 to the culture media, cells on the stiffest (77 kPa) hydrogels expressed the highest ALP activity, comparable to cells on tissue culture polystyrene (TCPS). ALP activity decreased as stiffness decreased, with the lowest expression observed on the softest (0.8 kPa) hydrogels. These results confirm previous reports that BMP‐2‐induced osteogenic differentiation in C2C12s and MSCs is mediated by matrix stiffness.^[^ ^17^, ^18^ ^]^

To gain more insight into the potential role of mechanotransduction in BMP‐2 signaling, C2C12 cell adhesion on hydrogels with varying stiffness was investigated. As anticipated from previous studies,^[^ ^21^, ^26^ ^]^ a higher amount of cells adhered on stiff hydrogels than on soft hydrogels (Figure 1e,f). Cell spread area was also greater on stiffer hydrogels (Figure 1g,h). BMP‐2 has been reported to increase cytoskeletal tension and alter spread area;^[^ ^27^ ^]^ however, we did not observe significant differences in spread area as a result of BMP‐2 treatment, suggesting that substrate stiffness overrides the effect of the growth factor. F‐actin staining showed that stress fibers were more pronounced and parallel to the cell's major axis on 35 and 77 kPa hydrogels, while cells on 8 kPa hydrogels were smaller and displayed decreased stress fiber bundles. On 0.8 kPa hydrogels, in which cells were the smallest, stress fiber formation was greatly hindered (Figure 1g). As stress fiber formation contributes to the generation of intrinsic cytoskeletal tension and is thus a key step in mechanotransduction, we can conclude that mechanotransduction may be linked to BMP‐2 signaling via cytoskeletal tension.

To confirm this, pharmacological agents that interfere with cellular tension and actin dynamics were utilized in cells on TCPS. As myosin is one of the key components regulating cytoskeletal tension, we employed blebbistatin (Blebb), an inhibitor of myosin II, and calyculin A (Calyc), which inhibits myosin light‐chain phosphatase from dephosphorylating myosin, resulting in increased myosin II activation. Perturbation of myosin II activity by Blebb dramatically decreased stress fiber formation and cell spread area (Figure 1g,h), as well as ALP activity (Figure 1b,d). In contrast, increased myosin II activation resulted in enhanced organization of stress fibers and cell spread area, as well as ALP activity. Surprisingly, stimulation by Calyc alone in the absence of BMP‐2 was sufficient to induce an increase in ALP expression in C2C12 cells. This is likely due to the largely enhanced cytoskeletal tension. Of all culture conditions, cells treated with both Calyc and BMP‐2 exhibited the highest ALP activity. Together, the results of the inhibitor experiments indicate that cytoskeletal tension is the mediator of BMP‐2‐induced osteogenic differentiation of C2C12 cells. This finding further raises the question of whether cytoskeletal tension is critical for either the initiation or the subsequent propagation of the BMP‐2 signaling cascade.

Elasticity directs cellular mechanotransduction through a set of extra‐ and intracellular signaling pathways involving integrins, focal adhesion kinase (FAK), Rho/ROCK, lamin‐A/C, and YAP/TAZ, to name a few.^[^ ^28^, ^29^ ^]^ As mechanosensitive signaling pathways have been heavily investigated in recent years, we chose here to focus specifically on the relationship between mechanosensitive signaling and BMP‐2 signaling.

### 2.2 BMP‐2 Signaling Initiation Is Relatively Independent of Mechanical Cues

Fusion and differentiation of C2C12 cells into multinucleated myotubes was monitored by staining for the marker myosin heavy chain (MHC).^[^ ^11^ ^]^ In the presence of BMP‐2, C2C12 cells failed to form MHC positive myotubes on TCPS or hydrogels, with or without pharmacological agent treatment (Figure S1, Supporting Information). Conversely, in the absence of BMP‐2, MHC‐positive myotubes were clearly observed when cells were confluent in all experimental conditions except Calyc treatment. This indicates that BMP‐2 signaling is initiated (as proven by inhibition of myogenesis), but later blocked (as proven by lack of osteogenesis), on soft hydrogels where ALP activity is at a minimum. To determine how mechanical cues affect BMP‐2 signaling, we assayed the BMP‐2‐induced Smad signaling cascade in a stepwise fashion.

**Figure 2:**
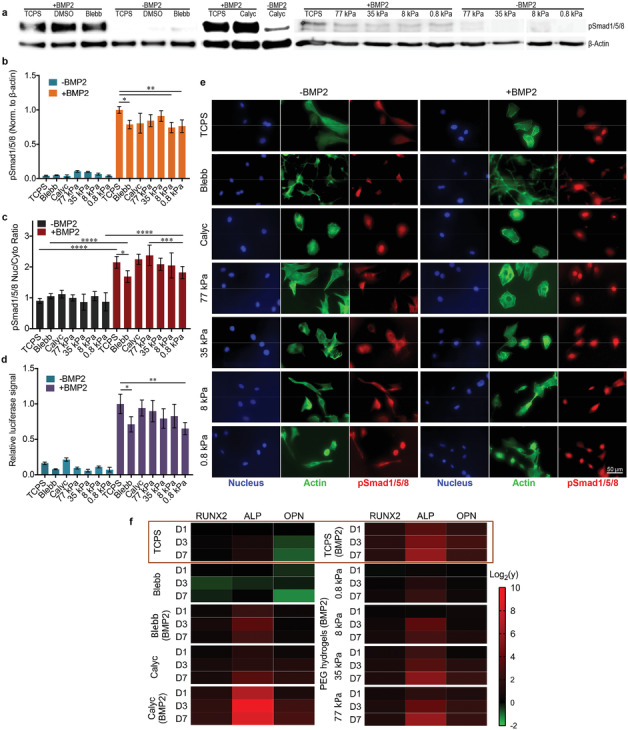
Activation of BMP‐2‐induced Smad signaling. a) Representative Western blots for pSmad1/5/8 and the housekeeper protein β‐actin of C2C12 cells that were stimulated for 60 min in different conditions before lysis. b) Quantification of Smad1/5/8 phosphorylation based on Western blot images; *n* = 1–2, two technical replicates. c) Nuclear‐to‐cytoplasmic ratios of pSmad1/5/8 for C2C12 cells cultured in different conditions for 3 h followed by BMP‐2 treatment for 1 h. Quantification was based on immunofluorescence images; *n* = 15, two technical replicates. d) Luciferase activity of C2C12 BRE‐Luc cells cultured in different conditions for 24 h; *n* = 2, three technical replicates. e) Representative immunofluorescence images of C2C12 cells stained with DAPI (blue), Alexa Fluor 488 phalloidin (green), and anti‐pSmad1/5/8 (red) after culturing in different conditions for 3 h followed by BMP‐2 treatment for 1 h. High‐magnification images of anti‐pSmad1/5/8 are available in Figure S2, Supporting Information. f) Heat map of the relative expression levels (y) of the genes related to the early (*RUNX2*), intermediate (*ALP*), and late (*OPN*) periods of osteogenesis in C2C12 cells cultured in different conditions. Data are analyzed by one‐way ANOVA followed by post hoc Tukey's multiple comparisons test for all panels.

BMP‐2 binds to and stabilizes membrane complexes consisting of types I and type II receptors. The type II receptor then phosphorylates and activates the type I receptor, recruiting and phosphorylating Smad1/5/8 at its C‐terminal SSXS‐motif to initiate the canonical Smad cascade.^[^ ^14^ ^]^ Thus, phosphorylated Smad1/5/8 (pSmad1/5/8) was used as a reporter for BMP‐2 activity.^[^ ^30^, ^31^ ^]^ To examine its phosphorylation state, cells were first seeded on TCPS or hydrogels with or without Blebb or Calyc pretreatment, then stimulated with BMP‐2 for 60 min. pSmad1/5/8 levels were determined by Western blot and normalized to β‐actin expression (**Figure** **2**a,b). In the absence of BMP‐2 stimulation, Smad1/5/8 phosphorylation was not observed in any experimental conditions. In BMP‐2 stimulated cells, the use of soft hydrogels or Blebb treatment resulted in a 20–25% decrease in Smad1/5/8 phosphorylation compared to TCPS or stiff hydrogels.

Downstream Smad signaling processes, which involve nuclear translocation of Smad complexes and further binding to target genes, were investigated by immunostaining of the Smad1/5/8 complex (Figure 2c,e) and luciferase reporter assay (Figure 2d), respectively. pSmad1/5/8 was highly enriched only in the nuclei of cells stimulated with BMP‐2, with Blebb treatment and the lowest hydrogel stiffness (0.8 kPa) inducing a slight decrease in nuclear localization. C2C12 cells stably transfected with a BMP‐responsive luciferase reporter (BRE‐Luc) containing Smad binding elements derived from the Id1 promoter^[^ ^32^ ^]^ were treated with BMP‐2. BRE‐Luc activity was fivefold to 10‐fold higher in cells stimulated with BMP‐2 compared to unstimulated cells. Consistent with Smad1/5/8 phosphorylation and Smad complex nuclear translocation, Blebb treatment or 0.8 kPa hydrogel stiffness resulted in a 30% decrease in BRE‐Luc activity.

It has been reported that cell shape regulates Smad signaling via RhoA/ROCK activity and downstream cytoskeletal tension generation. Smad signaling was decreased, but not totally blocked, when cell spreading was limited.^[^ ^15^ ^]^ Furthermore, cell spreading on matrix‐bound BMP‐2 has been shown to not affect Smad signaling; ROCK‐dependent cytoskeletal tension is not directly required for the Smad activation.^[^ ^21^ ^]^ On soft substrates, soluble BMP‐2 has been shown to decrease Smad activity to a certain degree. In our system, we also found that decreased cytoskeletal tension resulting from Blebb treatment or a soft substrate slightly decreased Smad signaling in cells stimulated with BMP‐2. However, Smad signaling in low tension, BMP‐2 stimulated cells was much higher than in unstimulated cells of all conditions. Thus, the initiation of the BMP‐2 signaling cascade via Smad signaling is relatively independent of mechanical cues.

### 2.3 Osteogenic Gene Expression Is Regulated by Mechanical Cues

As the activation of Smad signaling was found to be independent of mechanical cues, osteogenic gene expression was analyzed via quantitative real‐time reverse transcriptase polymerase chain reaction (qRT‐PCR). Three marker genes related to early (Runt‐related transcription factor 2; *RUNX2*), intermediate (*ALP*), and late (osteopontin; *OPN*) stages of osteogenic differentiation were assayed. All three markers were significantly upregulated in a time‐dependent fashion in C2C12 cells grown on TCPS and stiff 35 and 77 kPa hydrogels in the presence of BMP‐2 after 7 days (Figure 2f). The most dramatic upregulation of osteogenic markers was observed in cells cotreated with BMP‐2 and Calyc. Furthermore, Calyc treatment resulted in upregulation of these markers even in the absence of BMP‐2. In contrast, BMP‐2‐treated cells in combination with Blebb or cultured on the softest 0.8 kPa hydrogels displayed a reduction in osteogenic marker expression.

These patterns of osteogenic gene expression are consistent with the ALP activity assay and staining results described above. This observation suggests that osteogenic gene activation is mediated by cytoskeletal tension and dictated by mechanotransduction pathways. Thus, on soft matrices, the BMP‐2 signaling pathway driving osteogenic differentiation is blocked before gene expression is activated, although the Smad complex successfully binds to target genes (Figure 2d). Therefore, other transcription factors or transcriptional regulators may be required to coactivate osteogenic genes and promote subsequent cell fate specification.

### 2.4 YAP/TAZ Are Regulated by Cytoskeletal Tension

A wide range of transcription factors and transcriptional regulators play a role in mechanosensitive gene expression. Specifically, YAP and TAZ have been shown to be sensitive to mechanical cues via regulation of the Ras‐related GTPase RAP2^[^ ^33^ ^]^ and the ARID1A‐containing SWI/SNF complex.^[^ ^34^ ^]^ In addition, YAP/TAZ are well‐known for mediating cellular mechanoresponses.^[^ ^35^ ^]^

Thus, we investigated the correlation between endogenous YAP/TAZ subcellular localization and the presence of cytoskeletal tension in the presence or absence of BMP‐2 stimulation (Figures S3 and S4, Supporting Information). To this end, immunostaining of both YAP and TAZ was performed in C2C12 cells cultured on surfaces with different stiffness or treated with pharmacological inhibitors. YAP and TAZ both localized to the nucleus on rigid TCPS and stiffer 35 and 77 kPa matrices. On softer 0.8 and 8 kPa matrices, YAP and TAZ became predominantly cytoplasmic. Similarly, both YAP and TAZ were predominantly cytoplasmic when cytoskeletal tension was decreased via Blebb treatment. Calyc treatment did not alter YAP/TAZ localization for cells on TCPS, while BMP‐2 stimulation did not alter YAP/TAZ localization in any conditions.

Next, we investigated whether cell spread area regulates BMP‐2 signaling and YAP/TAZ localization. Micropatterned “islands” of defined size generated on an antifouling PEG coating were utilized to induce changes in cell spread area based on the available adhesive area in the presence of BMP‐2 (Figure S5, Supporting Information). On these micropatterns, nuclear translocation of Smad complexes was not affected by cell size. In contrast, YAP exhibited strong nuclear localization on large islands and weak nuclear localization on small islands. Similarly, osteogenic differentiation, as measured by osterix expression and localization, was enhanced on large islands. These results on single‐cell micropatterns rule out the effects of cell–cell contacts on BMP‐2 signaling pathways.^[^ ^8^ ^]^ YAP/TAZ localization in response to cell spreading and cytoskeletal tension support the notion that they are potential mediators of BMP‐2 signaling during osteogenic differentiation.

### 2.5 YAP/TAZ Regulate BMP‐2 Signaling

**Figure 3:**
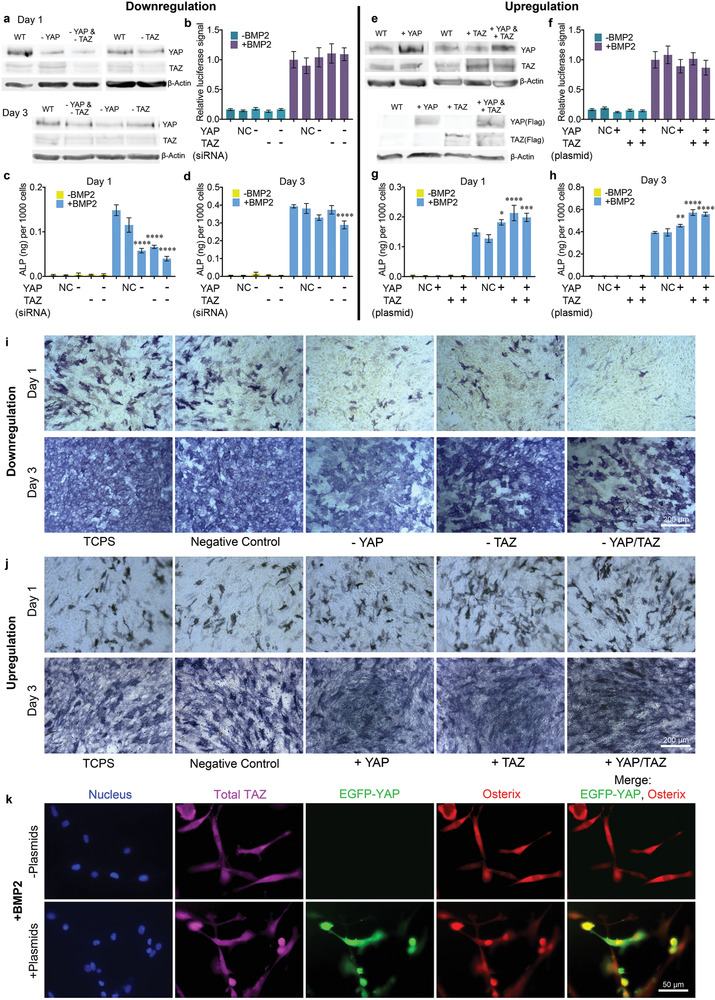
Upregulation and downregulation of YAP/TAZ to mediate BMP‐2 signaling. a) Representative Western blots of YAP and TAZ expression in lysates collected 1 or 3 days post‐siRNA treatment. b) Luciferase activity in C2C12 BRE‐Luc cells treated with siRNAs; *n* = 3. c,d) Quantification of ALP activity for C2C12 cells cultured c) 1 day or d) 3 days post‐siRNA treatment; *n* = 3. e) Representative Western blots of total YAP/TAZ and Flag‐tagged YAP/TAZ expression in lysates collected 1 day post‐transfection. f) Luciferase activity in C2C12 BRE‐Luc cells transfected with Flag‐tagged YAP/TAZ; *n* = 3. g,h) Quantitative assay of ALP activity for C2C12 cells cultured g) 1 day or h) 3 days post‐transfection; *n* = 2, two technical replicates. i,j) Representative images of ALP staining in BMP‐2‐stimulated C2C12 cells cultured 1 or 3 days i) post‐siRNA or j) Flag‐tagged YAP/TAZ transfection. k) Representative immunofluorescence images of C2C12 cells cultured on soft 0.8 kPa hydrogels in the presence of BMP‐2 2 days after transfection with pEGFP‐C3‐hYAP1 and pEF‐TAZ‐N‐Flag plasmid. Blue: DAPI; magenta: anti‐TAZ; green: plasmid‐generated YAP; red: anti‐osterix. Data are analyzed by one‐way ANOVA followed by post hoc Tukey's multiple comparisons test, *p*‐values referenced to NC group.

YAP/TAZ subcellular localization patterns merely indicate that YAP/TAZ are molecular “readers” of cytoskeletal tension. To prove that YAP/TAZ are relevant in mediating BMP‐2 signaling, transient siRNA‐induced knockdowns of YAP, TAZ, or both YAP and TAZ in C2C12 cells were performed and verified by Western blot (**Figure** **3**a). Importantly, Smad signaling was not affected by YAP/TAZ knockdown as measured by luciferase reporter assay (Figure 3b). Individual YAP or TAZ knockdown resulted in a reduction of BMP‐2‐induced ALP activity by nearly 50% within 1 day of siRNA treatment, and simultaneous knockdown of both YAP and TAZ resulted in an even stronger reduction in ALP activity (Figure 3c,i). After 3 days, ALP activity increased in all samples, consistent with transient recovery of YAP/TAZ. While individual YAP and TAZ knockdown samples returned to control levels, dual YAP/TAZ knockdown samples still exhibited statistically significant reductions in ALP activity after 3 days (Figure 3d,i).

We also examined YAP and TAZ overexpression in C2C12 cells via Flag‐tagged plasmid transfection, both individually and in combination (Figure 3e). Plasmid‐induced YAP/TAZ overexpression did not affect Smad signaling (Figure 3f). Overexpression of YAP, TAZ, and YAP/TAZ in combination for 1 or 3 days resulted in significantly higher levels of ALP activity than in control groups (Figure 3g,h,j). TAZ overexpression resulted in higher levels of ALP activity than YAP expression, potentially due to the fact that TAZ is negatively regulated by YAP expression but not vice versa,^[^ ^36^ ^]^ although we did not observe a decrease of TAZ levels during YAP overexpression by Western blot. We also did not observe this phenomenon in the YAP knockdown experiments, possibly because the siRNA‐induced knockdown we employed was transient as opposed to the more permanent short hairpin RNA (shRNA) or CRISPR‐Cas9 systems utilized in the literature.^[^ ^36^ ^]^ As YAP/TAZ are involved in numerous signaling pathways in cells,^[^ ^37^ ^]^ permanent perturbation may cause undesired cell responses including apoptotic cell death.^[^ ^38^ ^]^

Overexpression of both YAP and TAZ not only resulted in enhanced osteogenic differentiation on TCPS, but also stimulated osteogenic differentiation on soft 0.8 kPa matrices in the presence of BMP‐2, as measured by osterix nuclear localization (Figure 3k). The EGFP‐YAP plasmid and the TAZ plasmid were mixed together prior to lipids preparation for transfection. Nuclear osterix localization (red) was greatly enhanced in efficiently transfected cells (green), although increased YAP and TAZ expression did not enhance nuclear accumulation (as measured by nuclear‐cytoplasmic ratio) of YAP/TAZ (Figure S6, Supporting Information) as observed via immunostaining of total TAZ (magenta) and overexpressed YAP (green). In comparison, nuclear osterix localization was not observed in BMP‐2‐treated cells without plasmid expression. Thus, YAP/TAZ overexpression in the presence of BMP‐2 is sufficient for C2C12 osteogenesis, even when cytoskeletal tension is limited due to soft substrate elasticity.

The spread area and stress fiber assembly of TCPS‐adherent cells were also monitored when the expression of both YAP and TAZ was downregulated or upregulated. No obvious differences were detected (Figure S7, Supporting Information). Thus, cytoskeleton tension is not altered during siRNA or plasmid treatment. Together, this indicates that YAP and TAZ play a major role in BMP‐2‐induced osteogenesis by serving as mechanosensitive mediators of BMP‐2 signaling.

### 2.6 Smad Complexes and YAP/TAZ Synergize to Activate Gene Expression

To explore the interplay between Smad complexes and YAP/TAZ during mechanosensitive gene activation, C2C12 cells were treated with BMP‐2 and Blebb, followed by whole transcriptome shotgun sequencing (RNA‐Seq) (Data 1, Supporting Information). Principal component analysis (PCA) of 5813 differentially expressed genes (DEGs) inherent to specific treatment combinations (*q* value < 0.05) and hierarchical clustering analysis identified transcriptional signatures sufficient to differentiate cell populations as a function of treatment (Figure S8, Supporting Information).

**Figure 4:**
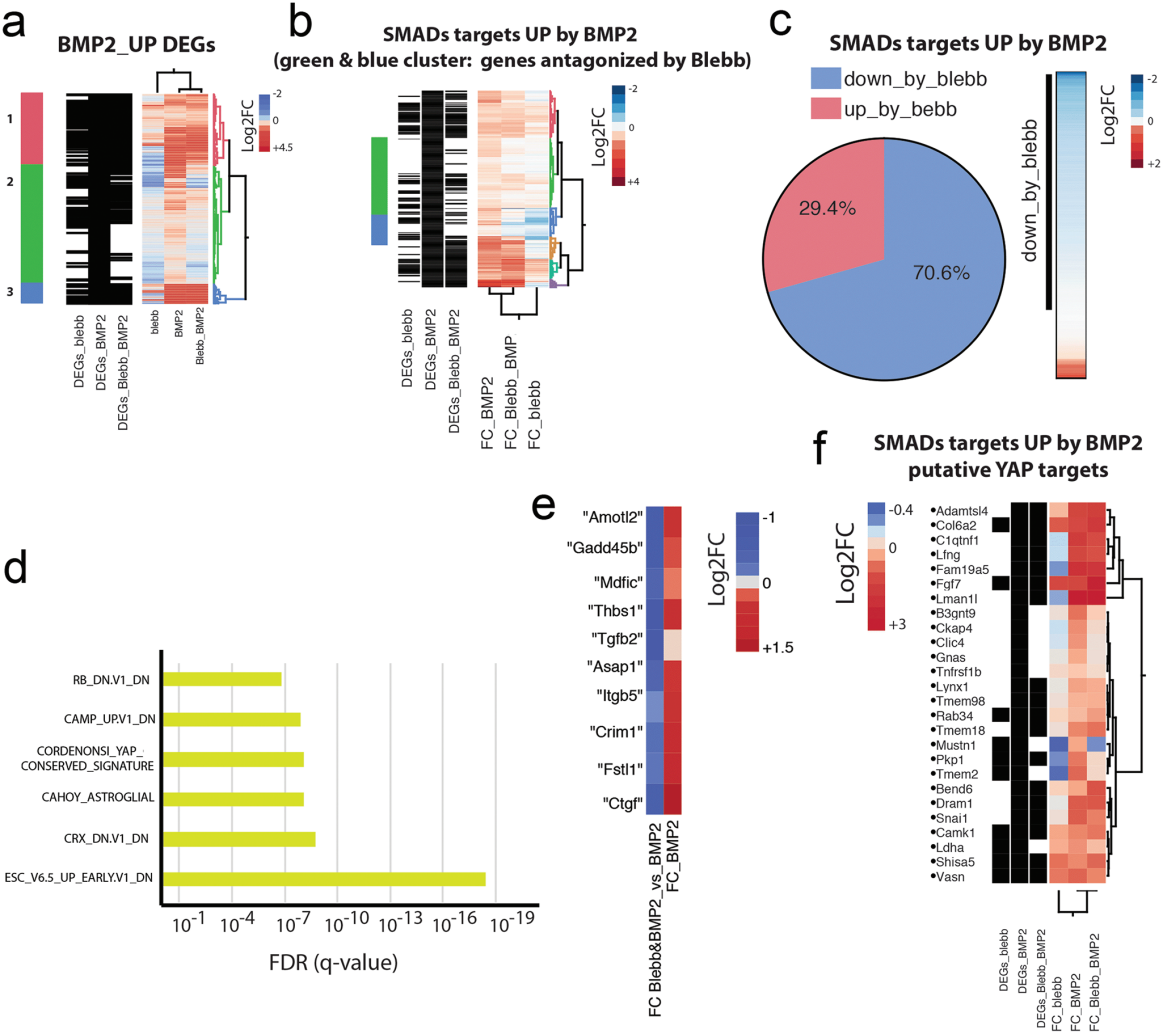
Cooperative gene activation by BMP‐2 stimulation and mechanical cues. a) Hierarchical clustering analysis of genes upregulated by BMP‐2. b) Hierarchical clustering analysis of the Smad1/4/5 targets identified from genes in (a). c) Pie chart of the Smad1/4/5 targets in (b) based on the data of Blebb&BMP‐2 versus BMP2. d) GSEA of BMP‐2 genes revealed an enrichment for YAP/TAZ‐regulated genes. e) Canonical YAP/TAZ targets were upregulated in C2C12 cells by BMP‐2 and antagonized by Blebb. f) Hierarchical clustering analysis of the YAP targets identified from genes in (b).

A total of 1536 genes were found to be significantly upregulated in response to BMP‐2 (Data 2, Supporting Information). Importantly, half of these BMP‐2 responsive genes (867 genes) were antagonized by Blebb, as revealed by unsupervised hierarchical clustering analysis (see green cluster, **Figure** **4**a), suggesting that this cluster of BMP‐2 responsive genes is mechanosensitive. Similarly, a subset of our BMP‐2 upregulated genes, which we characterized as bona fide Smad1/4/5 targets based on previous ChIP‐seq data^[^ ^39^ ^]^ (Data 3, Supporting Information), was significantly downregulated when BMP‐2 was coupled with Blebb (70% of these genes were antagonized by Blebb), indicating that the majority of Smad1/4/5 targets are also mechanosensitive (Figure 4b,c).

Gene set enrichment analysis (GSEA) of BMP‐2 genes revealed an enrichment of YAP/TAZ regulated genes (Cordenonsi signature, Figure 4d), suggesting a BMP‐2‐dependent regulation of YAP/TAZ targets. Indeed, canonical YAP/TAZ targets were upregulated in C2C12 cells by BMP‐2 and antagonized by Blebb (Figure 4e). Interestingly, 26 of our BMP‐2‐upregulated Smad1/4/5 targets have also been previously identified as targets of YAP,^[^ ^40^ ^]^ although in that study, BMP‐2 was not used to stimulate cells, suggesting that other Smad targets may also be YAP targets (Data 4, Supporting Information). BMP‐2‐induced upregulation of over 65% of these YAP target genes was decreased in response to Blebb treatment (Figure 4f), further strengthening the finding that BMP‐2‐induced gene expression is highly mechanosensitive.

These results suggest that 1) Smad complexes are capable of activating certain genes in the absence of mechanical stimulation, and 2) Smad complexes coactivate other genes in a concerted manner along with YAP/TAZ. The genes identified in (1) likely contribute to the inhibition of myotube formation, while those in (2) play a greater role in initiating osteogenic differentiation of C2C12 cells. In addition, osteogenic differentiation likely also includes other YAP/TAZ‐binding transcription factors. Indeed, the genes Id1, Id2, Id3, and Id4, all of which are DEGs upregulated by BMP‐2, were not affected by Blebb treatment (Figure S9, Supporting Information). These genes encode DNA‐binding protein inhibitors that have been shown to bind to and deactivate MyoD and its cofactors, resulting in an inhibition of myogenesis.^[^ ^41^ ^]^ Together, these results indicate that Smad signaling can occur both independent of and in conjunction with mechanical stimulation.

### 2.7 Nuclear Accumulation of Smad Complexes Maintains BMP‐2 Signaling after BMP‐2 Withdrawal but Additional Mechanical Stimulation Is Required for Osteogenesis

**Figure 5:**
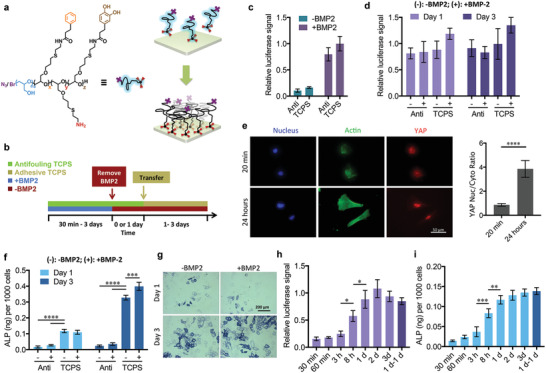
Synergy between BMP‐2 signaling and mechanotransduction. a) Antifouling coating scheme on TCPS. b) Scheme of the experimental setup. C2C12s were cultured on antifouling TCPS (green) with BMP‐2 stimulation (blue) for 30 min to 3 days. BMP‐2 was removed (red) and C2C12s were maintained in culture on antifouling TCPS for 1 day. C2C12s were subsequently transferred onto adhesive TCPS (yellow) for 1–3 days. c) C2C12 BRE‐Luc cell luciferase activity when cultured on antifouling or adhesive TCPS for 1 day; *n* = 3, two technical replicates. d) C2C12 BRE‐Luc cell luciferase activity when pretreated with BMP‐2 on antifouling TCPS for 1 day and then transferred onto antifouling or adhesive TCPS in the presence (+) or absence (–) of BMP‐2; *n* = 3, two technical replicates. e) Representative immunofluorescence images of BMP‐2‐pretreated (1 day) C2C12 cells stained with DAPI (blue), Alexa Fluor 488 phalloidin (green), and anti‐YAP (red) after being transferred onto adhesive TCPS for 20 min or 24 h, and related nuclear‐to‐cytoplasmic ratios of YAP; *n* = 20, Welch's *t*‐test. High‐magnification images of anti‐YAP are available in Figure S13, Supporting Information. f) Quantitative ALP activity assay for C2C12 cells pretreated with BMP‐2 on antifouling TCPS for 1 day and then transferred onto antifouling or adhesive TCPS in the presence (+) or absence (–) of BMP‐2; *n* = 3. g) Representative images of ALP staining for C2C12 cells pretreated with BMP‐2 on antifouling TCPS for 1 day and then transferred onto adhesive TCPS in the presence or absence of BMP‐2. h) Luciferase activity of C2C12 BRE‐Luc cells and i) ALP activity of C2C12 cells pretreated with BMP‐2 on antifouling TCPS from 30 min to 3 days, and then transferred onto adhesive TCPS without BMP‐2 for 1 day. For “1 d‐1 d” samples, cells were pretreated with BMP‐2 on antifouling TCPS for 1 day, and then cultured for an additional day on antifouling TCPS without BMP‐2, before being transferred onto adhesive TCPS without BMP‐2 for a final day; *n* = 4 for luciferase activity and *n* = 3 for ALP activity. The cell proliferation was limited by mitomycin C. Data are analyzed by one‐way ANOVA followed by post hoc Tukey's multiple comparisons test (except in (e), Welch's *t*‐test).

Data presented, thus, support the hypothesis that mechanical cues mediate BMP‐2 signaling, as osteogenic differentiation of C2C12 cells requires both Smad complex activation and YAP/TAZ localization. Next, we wanted to determine whether we could activate these two signaling pathways independently. We generated an antifouling polymer coating on TCPS based on a biomimetic amphiphilic block copolymer (**Figure** **5**a),^[^ ^42^ ^]^ which prevents ECM protein adsorption and cell adhesion,^[^ ^43^ ^]^ limiting external mechanical cues and cytoskeletal tension. C2C12 cells were initially stimulated with BMP‐2 on antifouling TCPS (Anti) and then transferred onto adhesive TCPS (untreated) without further BMP‐2 stimulation (Figure 5b). Cell adhesion was not observed on antifouling TCPS, but cells became well‐spread after transfer onto adhesive TCPS (Figure S10, Supporting Information). It is worth emphasizing that nonadherent cells were transferred via simple pipetting, allowing us to avoid trypsinization or other confounding factors present during passaging. Therefore, in this setup, Smad signaling can be activated first via BMP‐2 stimulation, followed by subsequent and separate mechanical stimulation and cytoskeletal tension induced by adhesive TCPS.

To eliminate the effects of proliferation, cells were pretreated with mitomycin C when cultured on adhesive TCPS for more than 1 day. Luciferase reporter assays indicated that C2C12 BRE‐Luc cells on antifouling TCPS exhibited slightly lower luciferase activity compared to cells on adhesive TCPS after BMP‐2 stimulation for 1 day (pretreatment), but exhibited eight times higher activity compared to cells that did not receive BMP‐2 treatment (Figure 5c). Interestingly, luciferase activity can be maintained in pretreated cells for at least 3 days on both antifouling and adhesive TCPS after the removal of BMP‐2 (Figure 5d). This means that BMP‐2‐induced Smad complexes continuously bind and activate target genes once initiation has occurred. Immunostaining also confirmed that nuclear pSmad1/5/8 localization was persistent in pretreated cells once they had been transferred onto adhesive TCPS in the absence of BMP‐2 (Figure S11, Supporting Information).

Nuclear YAP accumulation did not occur in pretreated cells subsequently transferred onto adhesive TCPS and fixed after 20 min, indicating that the pretreatment phase did not induce YAP/TAZ translocation. Once cells were sufficiently spread with well‐organized actin stress fibers, nuclear YAP accumulation was observed (Figure 5e). Pretreated cells did exhibit high ALP activity after both 1 and 3 days post‐transfer onto adhesive TCPS, but not when kept on antifouling TCPS (Figure 5f,g). Osteogenic differentiation was also monitored via osterix nuclear localization. In line with nuclear accumulation of YAP, only well‐spread, pretreated cells exhibited high osterix localization (Figure S12, Supporting Information).

To determine the effect of the duration of BMP‐2 pulse stimulation on Smad signaling, we provided BMP‐2 stimulation to C2C12 and C2C12 BRE‐Luc cells at various time points between 30 min and 3 days before transferring them onto adhesive TCPS. Cells were then cultured for 1 day in the absence of BMP‐2, after which luciferase reporter and ALP activity assays were performed. Luciferase activity, which was low when pretreatment only lasted 30 min, increased gradually until a pretreatment length of 1 day, at which point luciferase activity became saturated (Figure 5h). ALP activity followed a similar pattern (Figure 5i). Thus, higher‐level gene activation caused by Smad complexes in early signaling events only results in higher ALP activity after the application of mechanical cues. When cell proliferation is inhibited by mitomycin C, 1 day of BMP‐2 stimulation is sufficient to activate osteogenic differentiation in C2C12 cells. Moreover, luciferase activity and ALP activity did not decrease when pretreated cells were kept on antifouling TCPS for an additional day without BMP‐2 stimulation before being transferred onto adhesive TCPS (Figure 5h,i). These results 1) highlight the role of mechanotransduction in BMP‐2‐induced osteogenic differentiation, and 2) prove that C2C12 osteogenic commitment can be initiated in a step‐wise process via independent pulsed BMP‐2 stimulation and mechanical cues. In other words, cells can “remember” BMP‐2 stimulation history via Smad complex gene targeting. Osteogenic differentiation is subsequently activated via nuclear YAP/TAZ translocation as a function of cytoskeletal tension.

Human MSCs are a useful model for mechanosensitive stem cell differentiation. Similar to our findings in C2C12 cells, we observed osteogenic differentiation of MSCs in response to stimulation with BMP‐2 and mechanical cues (Figures S14–S16, Supporting Information). As MSCs are widely used in regenerative medicine and hold great therapeutic potential, these results extend our findings to more translational conditions.

## 3 Discussion

BMPs are a potent class of growth factors that regulate the development of many organ systems in the body and play an especially important role in osteogenesis. BMP therapies are becoming promising alternatives to autografts, which are currently the gold standard for chronic bone defects, but remain limited by low availability as well as donor site pain and inflammation.^[^ ^16^, ^44^ ^]^ Dysregulation of BMP signaling has been shown to contribute to a number of pathological processes, including cancer and ectopic bone formation.^[^ ^45^, ^46^ ^]^ Thus, understanding how BMP‐2 regulates differentiation and manipulating BMP‐2 signaling are both critically important for both clinical regenerative medicine and the rational design of growth factor‐doped biomaterials.

**Figure 6:**
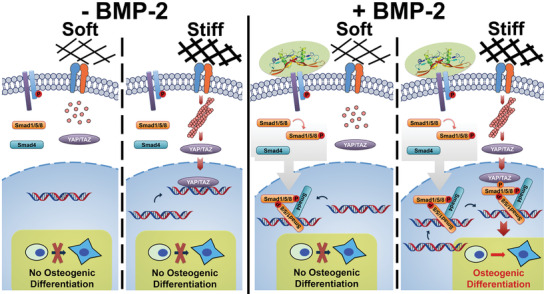
Synergy between cytoskeletal tension (YAP/TAZ) and the BMP‐2‐Smad pathway. Left: No activated Smad signaling or osteogenic differentiation is observed in the absence of BMP‐2, even in the presence of stiff matrix. Right: The initiation of BMP‐2‐induced Smad signaling is independent of cytoskeletal tension. Smad1/5/8 can be phosphorylated and form heteromeric complexes that translocate into the nucleus and bind to target genes. However, osteogenic gene activation requires cytoskeletal tension‐induced nuclear accumulation of YAP/TAZ. Thus, BMP‐2 signaling responds to mechanical cues by sensing nucleocytoplasmic shuttling of YAP/TAZ.

In this study, BMP‐2‐induced C2C12 osteogenesis was investigated from two complementary directions: 1) biochemical pathways that have been shown to play a role in BMP‐2 signaling, and 2) mechanotransduction pathways that are influenced by different matrix stiffness or the application of cytoskeletal tension‐modifying inhibitors. These synergistic modalities are summarized in **Figure** **6**. By transiently downregulating and upregulating the expression of YAP and TAZ in C2C12s, we found that they contribute to the crosstalk between BMP‐2 signaling and mechanotransduction pathways. Additionally, the shuttling of YAP and TAZ from the cytoplasm to the nucleus is independent of BMP‐2 signaling, but this translocation enhances BMP‐2‐induced differentiation. In parallel, the initiation of Smad signaling is independent of mechanical cues. Crosstalk between these two signaling pathways synergistically enhances osteogenic gene expression.

The crosstalk between BMP‐2 signaling and mechanotransduction pathways is likely due to binding between Smad heteromeric complexes and YAP/TAZ, possibly via phosphorylated Smad1,^[^ ^47^, ^48^ ^]^ as well as the assistance of other YAP/TAZ‐binding transcription factors. It has been suggested that TAZ coactivates RUNX2‐dependent gene transcription, driving cells towards osteogenic differentiation under BMP‐2 stimulation.^[^ ^49^ ^]^ In other studies focusing on TGF‐β signaling, the osteogenic gene activation is suggested to be coassociated with TEAD^[^ ^50^ ^]^ and OCT4.^[^ ^51^ ^]^ Thus, gene activation is likely the result of synergistic cooperation between multiple transcription factors regulated by diverse stimuli, as indicated by RNA‐seq analysis.

In 3D systems, YAP/TAZ may be regulated by mechanical cues in a different way than on 2D surfaces, likely due to spatial limitations on cytoskeletal assembly.^[^ ^52^ ^]^ For example, YAP/TAZ nuclear localization has been shown to be higher in cells encapsulated in soft hydrogels than in stiff ones.^[^ ^53^, ^54^ ^]^ Cells sense mechanical cues in both 2D and 3D conditions via the same mechanotransduction pathways. Thus, the key step in mechanotransduction in both 2D and 3D conditions is the generation of intracellular cytoskeletal tension. This tension is capable of inducing downstream YAP/TAZ nuclear localization and further crosstalk with BMP‐2 signaling pathways. In this work, we chose to focus on the intrinsic myosin‐based cytoskeletal tension generated on simple 2D material models, but it is reasonable to speculate that these same molecular mechanisms can be extended to 3D systems.

Smad signaling can be initiated by pulse BMP‐2 stimulation, which becomes saturated within 1 day. Interestingly, Smad complexes can continue binding to and activating target genes regardless of external mechanical cues after the removal of the BMP‐2 stimulus. This maintenance enables Smad‐activated cells to either keep their phenotype without applying further mechanical cues or differentiate to osteoblasts by applying later mechanical cues. The differentiation process is therefore programmable, a fact that can be leveraged in the future to control cells and growth factors in biomedical engineering. Please note the cell proliferation can dilute the concentration of targeted Smad complexes on genes. We only focused on the molecular mechanism and used mitomycin C to limit this dilution. For practical applications, we would suggest to initially treat cells with relatively high concentration of BMP‐2 for about 1 day and to keep the BMP‐2 concentration in relatively low level to continuously stimulate the proliferated cells. Furthermore, this sequential activation of differentiation programming may play a major role in cellular differentiation in vivo, where migrating stem cells are exposed to diverse and dynamic mechanical and biochemical environments on their journey from the stem cell niche to their ultimate differentiation site.

In addition to canonical Smad signaling, BMP‐2 also induces noncanonical pathways by activating MAPK cascades through Smad in a transcription‐independent fashion.^[^ ^55^, ^56^ ^]^ However, the molecular basis for the activation and signal transduction of noncanonical pathways is still not fully understood.

YAP/TAZ may not be the only crosstalk point between mechanotransduction pathways and BMP‐2 signaling. Integrins, which have been shown to colocalize with BMP‐2 receptors,^[^ ^57^ ^]^ may play a role in integrating mechanical signals with BMP‐2 signaling. Indeed, BMP‐2 stimulation has been shown to upregulate the expression of multiple types of αvβ‐ integrins in human osteoblasts.^[^ ^57^ ^]^ In addition, αvβ3 integrin mediates BMP‐2‐induced Smad signaling through the Cdc42‐Src‐FAK‐ILK cascade.^[^ ^21^ ^]^ Moreover, other cell membrane receptors like N‐cadherin^[^ ^58^ ^]^ and FGF receptor^[^ ^59^ ^]^ have been found to modulate BMP‐2. ROCK activity and RhoA/ROCK‐mediated cytoskeletal tension also regulate BMP‐induced Smad signaling and osteogenic differentiation in human MSCs.^[^ ^15^ ^]^ Although it has been demonstrated that mechanical cues affect Smad signaling through the various signaling pathways described above, here we demonstrate for the first time that cytoskeletal tension‐mediated nuclear accumulation of YAP/TAZ is critical for BMP‐2 signaling and subsequent osteogenic differentiation.

## 4 Conclusion

Overall, we used a carefully selected model cell line to study the crosstalk between BMP‐2 signaling and mechanotransduction pathways to understand how these two disparate modalities can synergize to mediate osteogenic differentiation at a molecular level. We identified YAP/TAZ as a primary crosstalk junction and demonstrated the integration of these two distinct pathways for altered gene transcription. In addition, Smad complexes, the transcription factors involved heavily in BMP‐2 signaling, were found to maintain BMP‐2 activity and remain bound to target genes after BMP‐2 withdrawal. This observation was then leveraged to dictate programmable BMP‐2 stimulation and mechanical cues for on‐demand cell fate determination. Investigations into the role of mechanotransduction in BMP signaling may identify important mechanisms linking chemical cues from the extracellular environment to physical cues from the ECM during cell differentiation and tissue development. Understanding this link will allow for the guided design of new biomaterials for regenerative therapies, as well as provide critical information on the use of BMP as a therapeutic tool for enhancing clinical bone repair success. Ultimately, future tissue engineering strategies for bone repair must take both biochemical and mechanobiological phenomena into account.

## 5 Experimental Section

##### PEG Hydrogels Fabrication and Mechanical Characterization

PEG hydrogel stiffness was controlled by varying poly(ethylene glycol)‐diacrylate (PEG‐DA) macromer with Mn 700 (455008 Sigma‐Aldrich) concentration in water. Specifically, 80, 100, 200, and 400 mg mL^–1^ concentrations were utilized. The adhesive peptide cyclo(Arg‐Gly‐Asp‐D‐Phe‐Cys) (c(RGDfC), PCI‐3686‐PI, Peptides International) was added to the PEG‐DA solution at a constant concentration of 100 × 10^−6^ m. The photoinitiator Irgacure 2959 (410896, Sigma‐Aldrich) was then mixed into the solution to achieve an initiator‐to‐acrylate ratio of 1:100. The gelation solution was pipetted onto glass coverslips modified with 3‐(trimethoxysilyl)propyl acrylate (475149, Sigma‐Aldrich) and covered with quartz slides modified with 1*H*,1*H*,2*H*,2*H*‐perfluorodecyltrichlorosilane (L1658403, Alfa Aesar). Polymerization was initiated via 365 nm UV irradiation. Fabricated hydrogels were equilibrated in phosphate‐buffered saline (PBS) buffer before use. For cell experiments, the c(RGDfC) peptide was utilized as an adhesive ligand. RGD peptide concentration was kept constant at 100 × 10^−6^ m, comparable to previous biological studies,^[^ ^60^ ^]^ and was sufficient to allow cells to adhere, but not induce spatial sensing‐induced cell adhesion.^[^ ^61^ ^]^ Mechanical properties of the hydrogels were measured with a rotational rheometer (Kinexus Pro, Malvern) in a humidity chamber at a constant shear rate of 1 Hz.

##### Cell Culture and Inhibitor Treatment

Mouse C2C12 myoblasts (ATCC CRL‐1772) were cultured as subconfluent monolayers in growth media, consisting of α‐MEM (A1049001, Gibco) supplemented with 10% fetal bovine serum (S0115, Biochrom) and 1% penicillin/streptomycin (15140122, Gibco) at 37 °C and 5% CO_2_.

Cells were serum‐starved in serum‐free growth media for 3–4 h before stimulation with 20 × 10^−9^ m recombinant human BMP‐2 (rhBMP‐2, Morphoplant GmbH) in growth media. For cells stimulated with BMP‐2 on antifouling surfaces, BMP‐2 was removed before cell transfer. Since cells could not adhere to the antifouling surface, their removal was possible with gentle pipetting. For this, media and cells were gently collected, centrifuged, and washed with PBS at least three times to remove residual exogenous BMP‐2 before seeding to new antifouling or cell adhesive TCPS.

For inhibitor studies, 20 × 10^−6^ m (–)blebbistatin (Blebb, B0560, Sigma‐Aldrich) or 1 × 10^−9^ m calyculin A (Calyc, C5552, Sigma‐Aldrich) was added to cell culture media. To prevent cell proliferation during Smad complex analysis post‐BMP‐2 removal, cells were treated with 10 µg mL^–1^ mitomycin C for 2 h before use.

##### ALP Assay

The SensoLyte pNPP alkaline phosphatase assay kit Colorimetric (AnaSpec) was used according to the manufacturer's instructions. Cell numbers were counted prior to lysis. Final values were measured at a wavelength of 405 nm with a Tecan Infinite M200 Plate Reader. ALP staining was performed with Leukocyte Alkaline Phosphatase kit based on Naphthol AS‐BI and fast blue BB salt (86C, Sigma‐Aldrich) according to manufacturer's instructions.

##### Protein Isolation and Western Blot Analysis

Cells were lysed in RIPA buffer (89900, Thermo Fisher Scientific) freshly supplemented with protease and phosphatase inhibitor cocktails (78442, Thermo Fisher Scientific) on ice and then centrifuged at 14 000 × *g* for 15 min at 4 °C. Lysates were collected and stored at –20 °C. After BCA assays to determine concentration, protein extracts were separated via SDS‐PAGE and transferred to a nitrocellulose membrane. After blocking with 5% BSA in TBST for 1 h, membranes were incubated overnight with diluted primary antibodies in 5% BSA in TBST at 4 °C followed by secondary HRP‐linked antibodies at room temperature for 2 h. Chemiluminescence via Amersham ECL Prime Western Blotting Detection Reagent (GE Healthcare) was detected with a Fujifilm LAS‐3000 Imager. ImageJ was used for band quantification.

For the Western blot analysis of pSmad1/5/8, more than 10 k cells were seeded on substrates in serum‐free growth media for 3 h before stimulation with 20 × 10^−9^ m BMP‐2 for 1 more hour. If required, 20 × 10^−6^ m Blebb or 1 × 10^−9^ m Calyc was added during the whole 4 h. For the Western blot analysis of YAP/TAZ, more than 10 k cells were treated as described below (Section RNA Interference and Plasmid Transfection).

Primary antibodies and corresponding concentrations used in this study were rabbit anti‐pSmad1/5/8(9) (1:1000) (13820, Cell Signaling Technology), rabbit anti‐YAP (1:1000) (4912, Cell Signaling Technology), mouse anti‐TAZ (1:1000) (560235, BD Pharmingen), mouse anti‐β‐actin (1:2000) (A1978, Sigma‐Aldrich), and mouse anti‐FLAG (1:1000) (F1804, Sigma‐Aldrich). Secondary antibodies were HRP‐linked anti‐rabbit IgG (1:2000) (7074, Cell Signaling Technology) and HRP‐linked anti‐mouse IgG (1:2000) (7076, Cell Signaling Technology). PageRuler Plus Prestained Protein Ladder, 10–250 kDa, served as a molecular weight marker (26619, Thermo Fisher Scientific).

##### Luciferase Assay

Luciferase assays were performed using the Luciferase Assay System (E1500, Promega) according to the manufacturer's instructions. C2C12 BRE‐Luc cells stably transfected with pGL3(BRE)‐luciferase reporter construct have been previously described.^[^ ^32^ ^]^ Cell numbers were counted prior to lysis. Chemiluminescence was detected with a Tecan Infinite M200 Plate Reader and normalized to cell counts.

##### Immunofluorescence Staining and Microscopy

Cells were washed once with cell culture media and twice with PBS before fixation with 4% paraformaldehyde at room temperature for 15 min. Samples were then washed thrice with ice cold PBS. Cells were permeabilized with 0.25% v/v Triton X‐100 in PBS for 10 min at room temperature, then washed thrice with PBS. Nonspecific antibody binding was blocked by incubating samples with 1% w/v bovine serum albumin (BSA) in PBST (0.1% v/v Triton X‐100 in PBS) at room temperature for 45 min. Next, samples were washed briefly with PBST and incubated with primary antibodies for 60 min at room temperature. Following primary antibody incubation, samples were washed twice with PBST and thrice with PBS. Samples were then incubated with secondary antibodies and Alexa Fluor 488 phalloidin for 60 min at room temperature, followed by washing twice with PBST and thrice with PBS. Finally, samples were placed on microscope slides in Fluoromount‐G with DAPI (00‐4959‐52, eBioscience) mounting media. Immunofluorescence images were acquired on an Axiovert 200M Microscope (Carl Zeiss).

Primary antibodies and corresponding concentrations used were rabbit anti‐pSmad1/5/8(9) (1:200) (13820, Cell Signaling Technology), rabbit anti‐YAP (1:100) (4912, Cell Signaling Technology), mouse anti‐TAZ (1:100) (560235, BD Pharmingen), rabbit anti‐Osterix (1:100) (ab22552, Abcam), and mouse anti‐myosin heavy chain (1:10) (MF20, Developmental Studies Hybridoma Bank, University of Iowa). Secondary antibodies used were Alexa Fluor 568‐linked anti‐rabbit IgG (1:1000) (A11011, Thermo Fisher Scientific), Alexa Fluor 647‐linked anti‐mouse IgG (1:1000) (A21235, Thermo Fisher Scientific), and Alexa Fluor 488‐linked anti‐mouse IgG (1:1000) (A11029, Thermo Fisher Scientific).

##### RNA Extraction and qRT‐PCR

Total RNA was isolated using TRIzol reagent (15596026, Invitrogen) according to the manufacturer's instructions. Collected RNA was converted to cDNA using a high‐capacity cDNA reverse transcription kit (4368814, Applied Biosystems) along with an iCycler thermal cycler gradient (Bio‐Rad) according to the manufacturer's instructions. Gene expression profiles were determined for RUNX2, ALP, and OPN with glyceraldehyde 3‐phosphate dehydrogenase used as a housekeeping gene. qRT‐PCR was carried out on a 7500 Real‐Time PCR System (Applied Biosystems). cDNA was amplified with the following conditions: 1 cycle at 50 °C for 2 min and 95 °C for 2 min, then 40 cycles at 95 °C for 15 s and 60 °C for 1 min. Amplification was monitored with SYBR Green (4309155, Applied Biosystems). Data were then normalized to the housekeeping gene as an index of cDNA content after reverse transcription and further normalized to the group on TCPS at day 1. Primer sequences^[^ ^62^ ^]^ are listed in the Table S1, Supporting Information.

##### RNA‐Seq

Total RNA from C2C12 cells was extracted with a Quick‐RNA miniprep kit from Zymo Research (R1055) following manufacturer's instructions. RNA concentration was assessed via NanoDrop and then stored at –80 °C.

For RNA‐Seq experiments, library preparation was performed with the TruSeq RNA Sample Prep Kits v2 (Illumina) following manufacturer's instruction. RNA‐Seq libraries were then run on the Agilent 2100 Bioanalyzer (Agilent high‐sensitivity DNA chip) for quantification and quality control and then sequenced on NovaSeq 6000 (Illumina).

##### RNA Interference

To knock down endogenous YAP and TAZ levels, C2C12 cells were transfected with siRNAs against YAP (TriFECTa DsiRNA Kit, design ID, mm.Ri.Yap1.13, Integrated DNA Technologies, Inc.) or TAZ (Stealth siRNAs MSS227747, MSS227748, MSS227749, Thermo Fisher Scientific) using Lipofectamine 2000 (11668027, Thermo Fisher Scientific) according to the manufacturer's instructions. To analyze resulting protein expression levels, cells were lysed for Western blot analysis at indicated time points after transfection.

Cells were seeded in antibiotic‐free growth media for 24 h prior to transfection. Transfection solution was prepared as follows: 1 mg mL^–1^ Lipofectamine 2000 was diluted to 30 µg mL^–1^ in Opti‐MEM media (31985, Gibco) and mixed for 15 min, while 20 × 10^−6^ m solutions of each siRNA were diluted 20× to 1 × 10^−6^ m in a separate aliquot of Opti‐MEM media. The two solutions were then mixed at a 1:1 by volume for another 15 min at room temperature. Afterwards, the final mixture was added into cell culture medium without antibiotics to achieve a final concentration of 100 × 10^−9^ m for each siRNA in 3 µg mL^–1^ Lipofectamine 2000. Cells were then incubated with siRNA for 24 h at 37 °C with 5% CO_2_. Cells treated with Lipofectamine 2000 alone were used as a negative control group.

##### Plasmid Transfection

p2xFLAGhYAP1 and pEGFP‐C3‐hYAP1 plasmids were provided by Marius Sudol (17791 and 17843, Addgene);^[^ ^63^, ^64^ ^]^ pEF‐TAZ‐N‐Flag plasmids were provided by Michael Yaffe (19025, Addgene).^[^ ^65^ ^]^ Plasmids were isolated and purified with a Qiagen Plasmid Plus Midi Kit (12943, Qiagen) and were stored at –80 °C.

Cells were seeded in antibiotic‐free growth media for 24 h prior to transfection. Transfection solution was prepared as follows: 1 mg mL^–1^ Lipofectamine 2000 was diluted to 60 µg mL^–1^ in Opti‐MEM media (31985, Gibco) and mixed for 15 min, while 200 ng µL^–1^ solutions of plasmid were diluted 10× to 20 ng µL^–1^ in a separate aliquot of Opti‐MEM media. The two solutions were then mixed at a 1:1 by volume for another 15 min at room temperature. Afterwards, the final mixture was added into cell culture media without antibiotics to achieve a final concentration of 1 ng µL^–1^ for each plasmid in 3 µg mL^–1^ Lipofectamine 2000. Cells were then incubated with plasmid for 24 h at 37 °C with 5% CO_2_. Cells treated with Lipofectamine 2000 alone were used as a negative control group.

##### Statistics

Statistical analysis was performed in GraphPad Prism 7. One‐way ANOVA followed by post hoc Tukey's multiple comparisons test or Welch's *t*‐test were carried out as described in figure captions. All results are displayed as mean ± standard deviation, *p < 0.05, **p < 0.01, ***p < 0.001, and ****p < 0.0001. Significance is indicated with by *(*p* < 0.05).

## Conflict of Interest

The authors declare no conflict of interest.

## Supporting information

Supporting Information

Supporting Information

Supporting Information

Supporting Information

Supporting Information
