# Supplementary material for: BMP‐2 Signaling and Mechanotransduction Synergize to Drive Osteogenic Differentiation via YAP/TAZ
Source: Adv Sci (Weinh). 2020 Jun 16;7(15):1902931. doi: 10.1002/advs.201902931 (PMC7404154; doi:10.1002/advs.201902931)
Supplement: Supplementary file 1 — Supporting Information [file ADVS-7-1902931-s001.pdf]

© 2020 Wiley-VCH GmbH

ADVANCED SCIENCE
Open Access

Supporting Information

for *Adv. Sci.*, DOI: 10.1002/advs.201902931

BMP-2 signaling and mechanotransduction synergize to drive osteogenic differentiation via YAP/TAZ

Qiang Wei, Andrew Holle, Jie Li, Francesca Posa, Francesca Biagioni, Ottavio Croci, Amelie S. Benk, Jennifer Young, Fatima Noureddine, Jie Deng, Man Zhang, Gareth J. Inman, Joachim P. Spatz, Stefano Campaner, Elisabetta A. Cavalcanti-Adam**

Supporting Information

BMP-2 signaling and mechanotransduction synergize to drive osteogenic differentiation via YAP/TAZ

Qiang Wei, Andrew Holle, Jie Li, Francesca Posa, Francesca Biagioni, Ottavio Croci, Amelie S. Benk, Jennifer Young, Fatima Noureddine, Jie Deng, Man Zhang, Gareth J. Inman, Joachim P. Spatz, Stefano Campaner, Elisabetta A. Cavalcanti-Adam**

Differentiation of C2C12 cells into myotubes

Fusion and differentiation of C2C12 cells into multinucleated myotubes was monitored by staining for the marker myosin heavy chain (MHC)[1]. In the presence of BMP-2, C2C12s failed to form MHC-positive myotubes on TCPS or hydrogels, with or without pharmacological agent treatment (Supplementary Fig. 1). Conversely, in the absence of BMP-2, MHC positive myotubes were clearly observed when C2C12 cells were confluent in all experimental conditions except Calyc treatment. This indicates that BMP-2 signaling is initiated but blocked at certain step in cells on soft hydrogels where ALP activity is at a minimum. (**Fig. 1**).

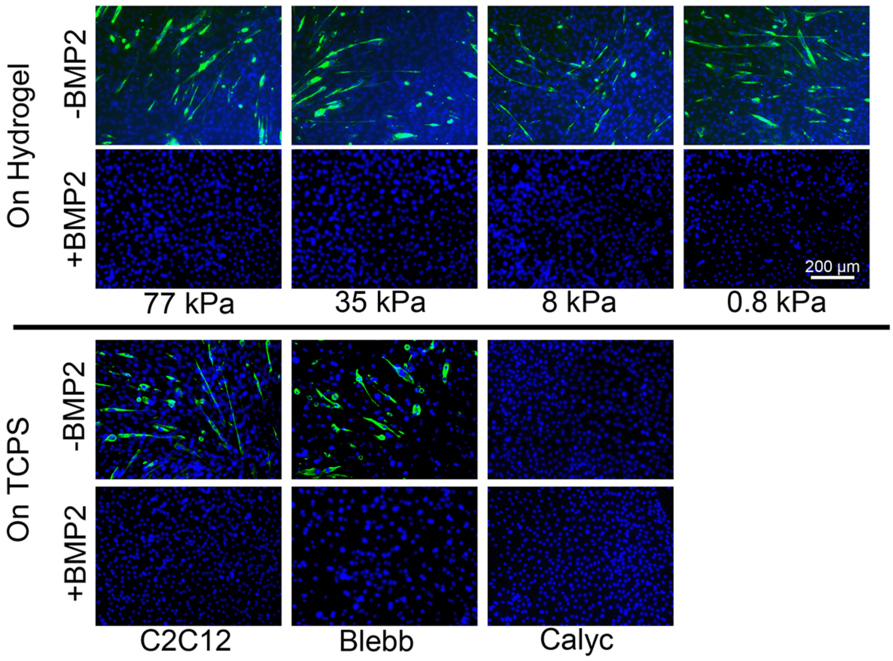

Supplementary Fig. 1 Representative DAPI (blue) and anti-Myosin heavy chain (green) immunofluorescence images of C2C12 cells cultured for 7 days in different conditions.

Supplementary Fig. 2 High magnification images of pSmad1/5/8 expression found in Figure 2e.

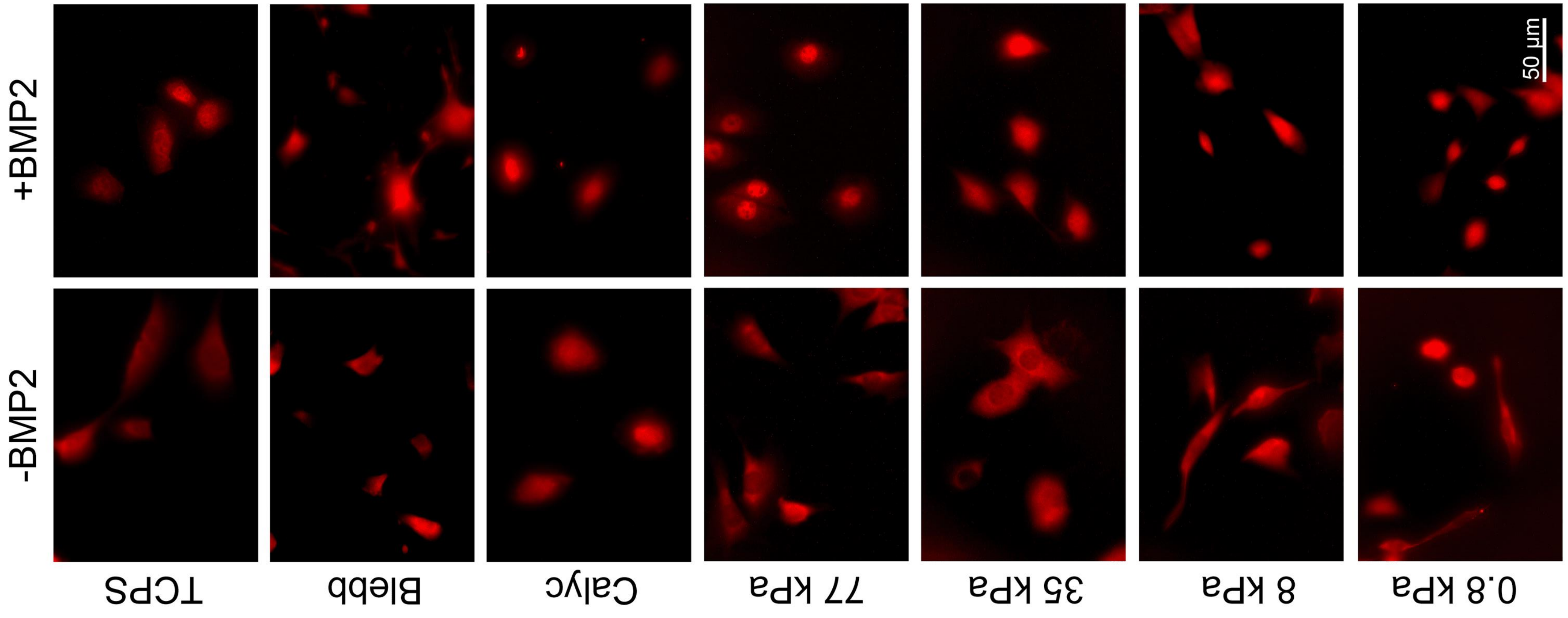

YAP/TAZ subcellular localization

We investigated the correlation between endogenous YAP/TAZ subcellular localization and the presence of cytoskeletal tension in the presence or absence of BMP-2 stimulation (Supplementary Fig. 3, 4). To this end, immunostaining of both YAP and TAZ was performed in C2C12 cells cultured on surfaces with different stiffness values or treated with pharmacological inhibitors. YAP and TAZ both localized to the nucleus on rigid TCPS and stiffer 35 kPa and 77 kPa matrices. On softer 0.8 kPa and 8 kPa matrices YAP and TAZ became predominantly cytoplasmic. Similarly, both YAP and TAZ were predominantly cytoplasmic when cytoskeletal tension was decreased *via* Blebb treatment. Calyc did not cause any difference in YAP/TAZ localization for cells on TCPS. BMP-2 stimulation did not significantly change YAP/TAZ localization in any conditions.

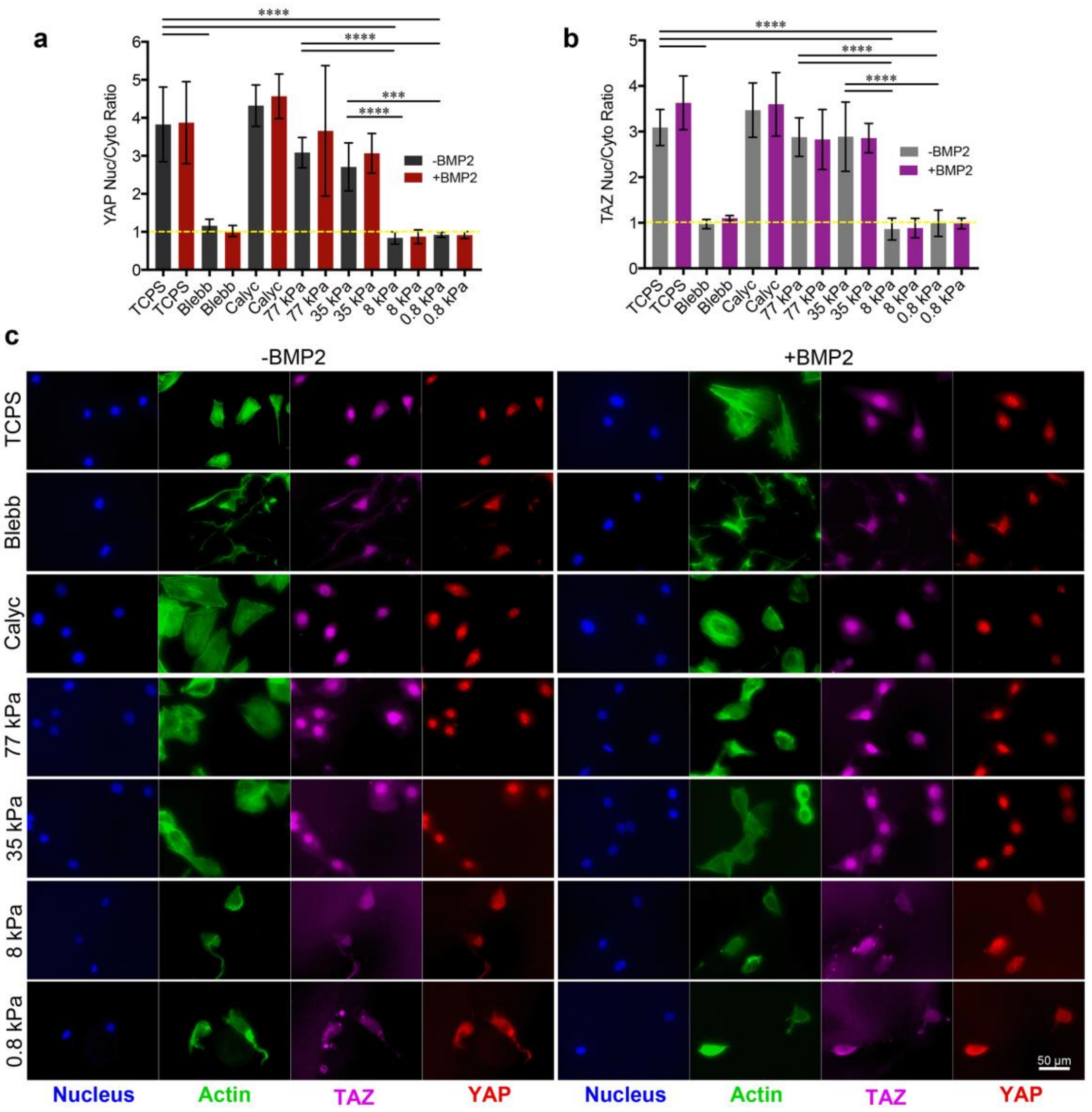

Supplementary Fig. 3 YAP/TAZ subcellular localization. **a** Nuclear-to-cytoplasmic ratios of YAP and **b** TAZ for C2C12 cells cultured in different conditions for 24 hours. Quantification was based on immunofluorescence images (n = 15, 2 technical replicates, one-way ANOVA followed by post hoc Tukey's multiple comparisons test). **c** Representative immunofluorescence images of C2C12 cells stained with DAPI (blue), Alexa Fluor 488 Phalloidin (green), anti-TAZ (magenta), and anti-YAP (red) after culturing in different conditions for 24 hours. High magnification images of anti-TAZ and anti-YAP are displayed in Supplementary Fig. 4.

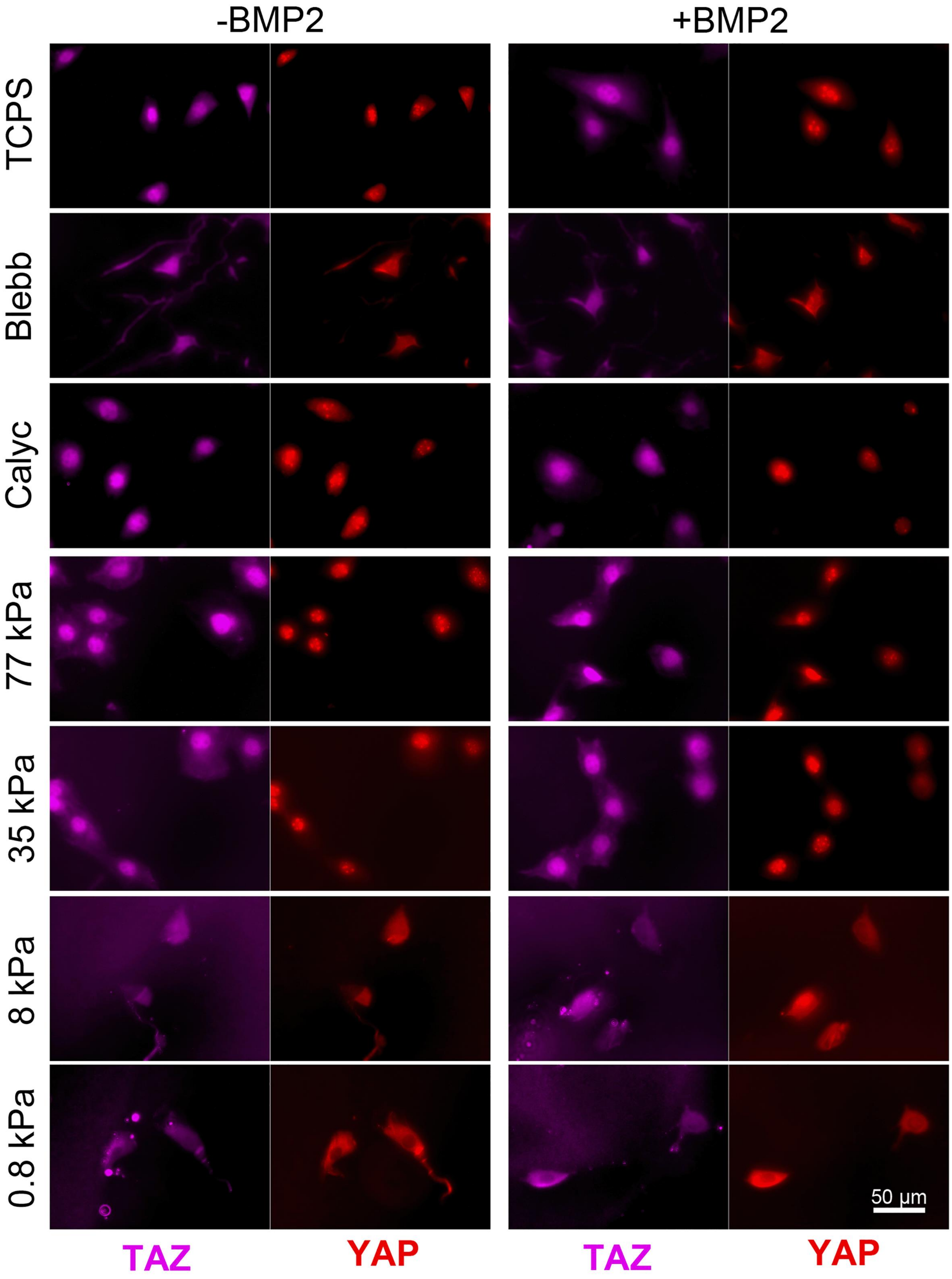

Supplementary Fig. 4 High magnification images of TAZ and YAP expression found in Supplementary Fig. 3c.

Cells with controllable spread area

We investigated whether cell spread area regulates BMP-2 signaling and YAP/TAZ localization. Micropatterned 'islands' of defined size generated on an antifouling PEG coating were utilized to induce changes in cell spread area based on the available adhesive area in the presence of BMP-2 (Supplementary Fig. 5). On these micropatterns, nuclear translocation of Smad complexes was not affected by cell size. In contrast, YAP exhibited strong nuclear localization on large islands and weak nuclear localization on small islands. Similarly, osteogenic differentiation, as measured by osterix expression and localization, was enhanced on large islands. These results on single-cell micropatterns rule out the effects of cell-cell contacts on BMP-2 signaling pathways[2].

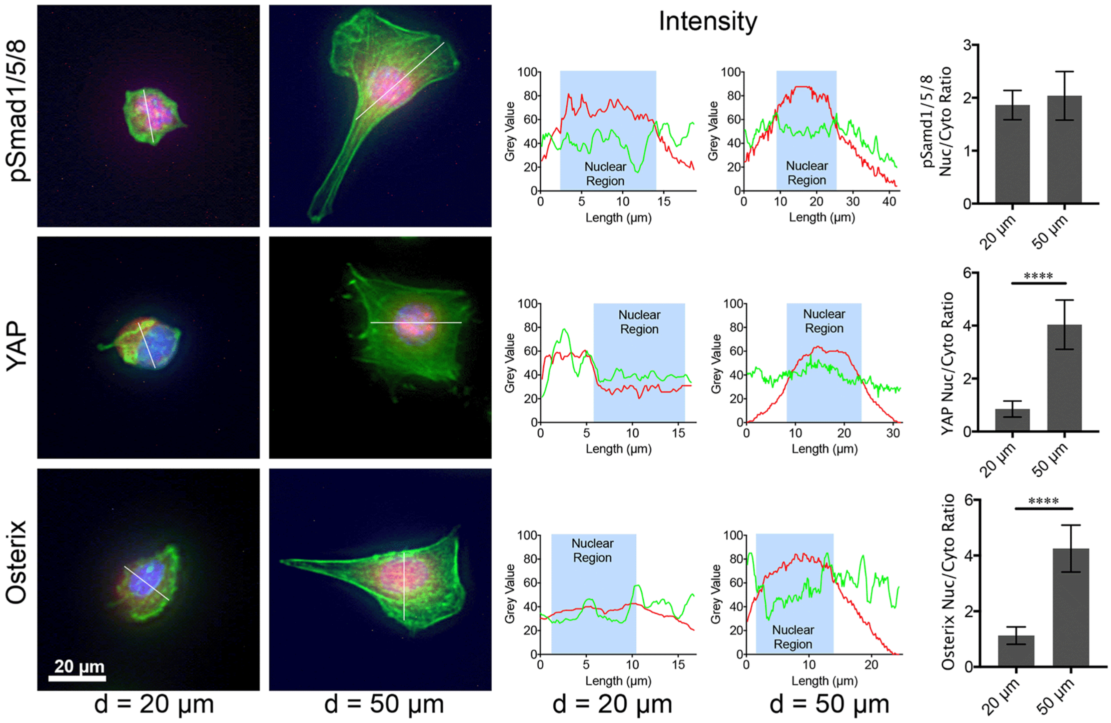

Supplementary Fig. 5 Representative immunofluorescence images and corresponding nuclear-to-cytoplasmic ratios of pSmad1/5/8, YAP, and osterix for C2C12 cells cultured on micropatterned 'islands' of 20 µm and 50 µm diameter in the presence of BMP-2 for 24 hours. Cells were stained with DAPI (blue), Alexa Fluor 488 Phalloidin (green), anti-pSmad1/5/8 (red, upper), anti-YAP (red, middle), and anti-Osterix (red, lower). For nuclear-to-cytoplasmic ratios, n = 10, one-way ANOVA followed by post hoc Tukey's multiple comparisons test.

YAP/TAZ regulation

The EGFP-YAP plasmid and the TAZ plasmid were mixed together prior to lipids preparation for transfection (**Fig. 3k**). The increased YAP and TAZ expression did not enhance the accumulation ratio (nuclear-cytoplasmic ratio) of YAP/TAZ (Supplementary Fig. 6) in cellular nuclei as indicated by the immunostaining of total TAZ (magenta) and overexpressed YAP (green).

The cell spread size and stress fiber assembly of the cells adhered on TCPS were also monitored when the expression of both YAP and TAZ was down- or upregulated. No obvious difference was detected (Supplementary Fig. 7). Thus, the cytoskeleton tension was not altered during this short time of siRNA or plasmid treatment.

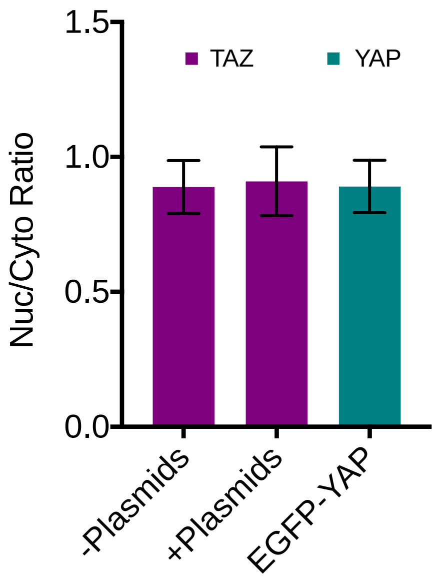

Supplementary Fig. 6 Nuclear-to-cytoplasmic ratios of total TAZ (column 1 and 2) and overexpressed YAP (column 3) of C2C12 cells cultured on soft 0.8 kPa hydrogels in the presence of BMP-2 2 days after transfection with pEGFP-C3-hYAP1 and pEF-TAZ-N-Flag plasmid (n = 8, 2 technical replicates). Representative immunofluorescence images were shown in Figure 3k.

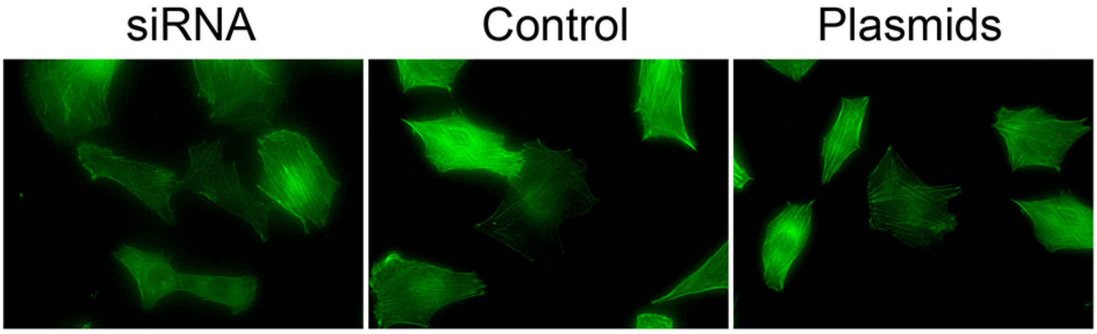

Supplementary Fig. 7 Representative images of F-actin staining of C2C12 cells cultured on TCPS for 1 day after transfection with siRNA or plasmid for both YAP and TAZ.

RNA-seq analysis

To explore the interplay between Smad complexes and YAP/TAZ during mechanosensitive gene activation, C2C12 cells were treated with BMP-2 and Blebb, followed by whole transcriptome shotgun sequencing (RNA-Seq) (Supplementary Data 1). Principal component analysis (PCA) (Supplementary Fig. 8a) and hierarchical clustering analysis (Supplementary Fig. 8b) identified transcriptional signatures sufficient to differentiate cell populations as a function of treatment, including 5,813 differentially expressed genes (DEGs) inherent to specific treatment combinations ($p<0.05$, Supplementary Fig. 8c).

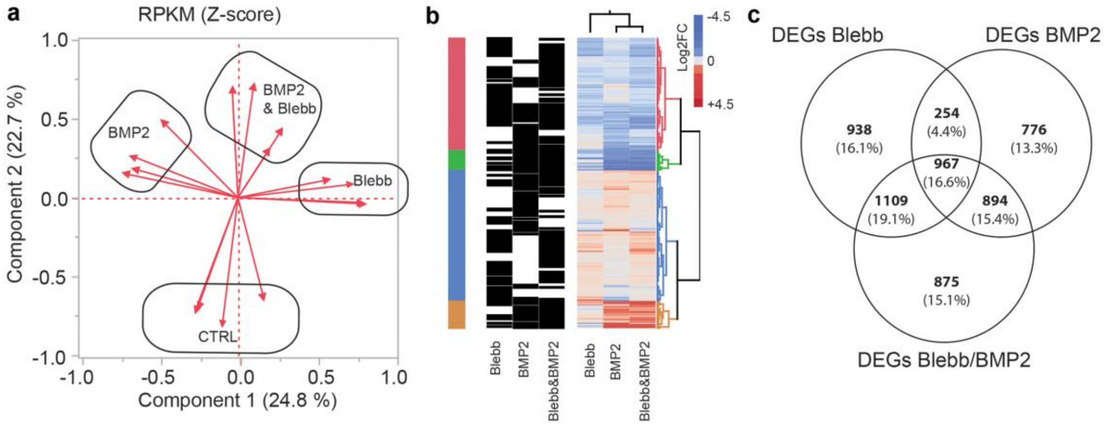

Supplementary Fig. 8 RNA-seq analysis. **a** Principal component analysis (PCA) for clustering gene expression data. **b** Hierarchical clustering for gene expression data analysis. **c** Venn diagram summarization of differential expression comparisons

Myogenesis related gene activation

As indicated by RNA-Seq analysis, the genes Id1, Id2, Id3, and Id4, all of which are DEGs upregulated by BMP-2, were not affected by Blebb treatment (Supplementary Fig. 9). These genes encode DNA-binding protein inhibitors which have been shown to bind to and deactivate MyoD and its cofactors, resulting in an inhibition of myogenesis.

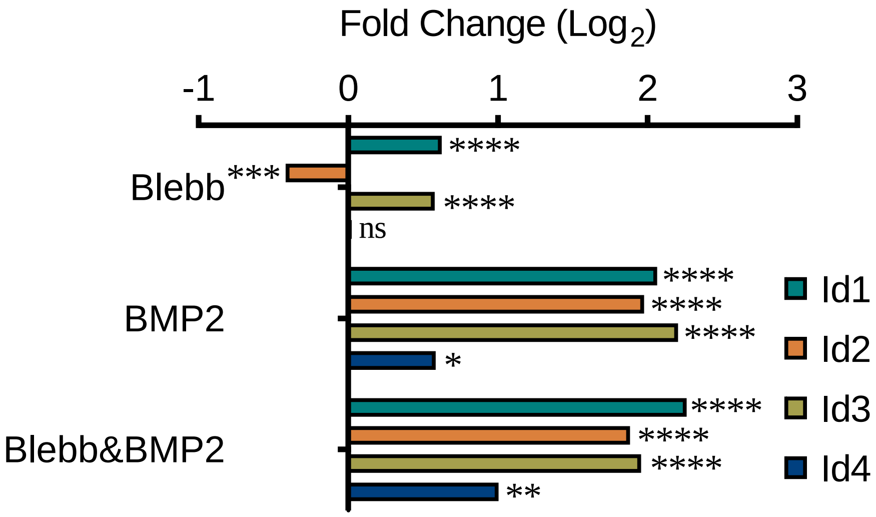

Supplementary Fig. 9 The expression level of ID1, ID2, ID3, and ID4 genes as indicated by RNA-Seq analysis.

Cell adhesion on antifouling polymer coatings

The generated antifouling polymer coating on TCPS (**Fig. 5a**) prevented the adhesion of C2C12 cells (Supplementary Fig. 10 left) to limit external mechanical cues and cytoskeletal tension. Cells were fully spread after transfer onto cell adhesive TCPS (Supplementary Fig. 10 right).

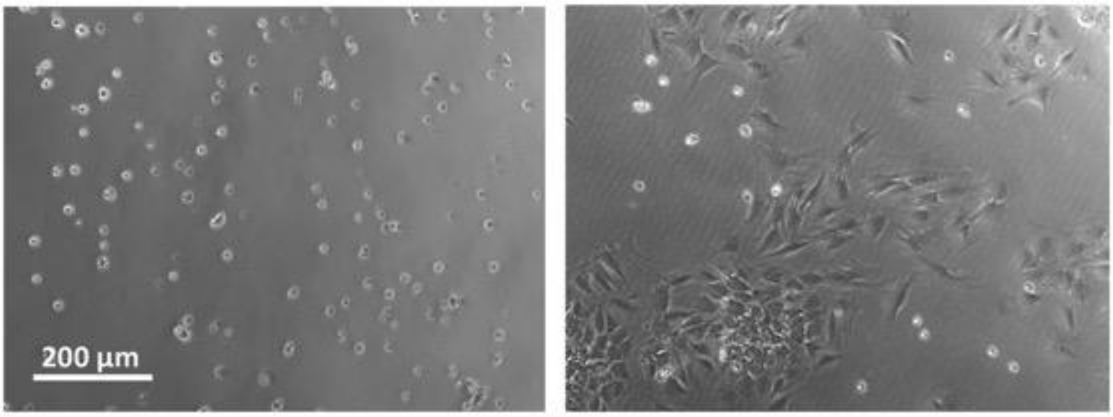

Supplementary Fig. 10 Cell adhesion on antifouling TCPS after 1 day in culture (left) and 1 day after being transferred onto cell adhesive TCPS (right)

Immunostaining for pSmad1/5/8

C2C12 cells were initially stimulated with BMP-2 on antifouling TCPS (Anti) and then transferred onto adhesive TCPS (non-treated TCPS) without further BMP-2 stimulation (**Fig. 5b**). Immunostaining confirmed that nuclear pSmad1/5/8 localization was maintained in pretreated cells once they had been transferred onto adhesive TCPS in the absence of BMP-2 (Supplementary Fig. 11).

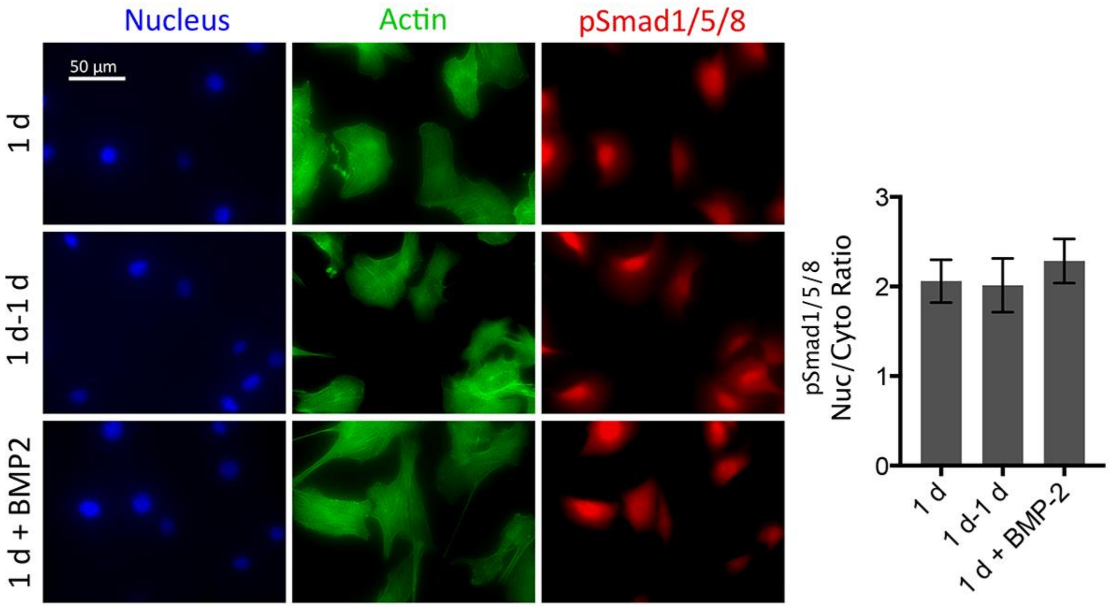

Supplementary Fig. 11 Representative immunofluorescence images and corresponding nuclear-to-cytoplasmic ratios of pSmad1/5/8 for BMP-2 pretreated C2C12 cells stained with DAPI (blue), Alexa Fluor 488 Phalloidin (green), and anti-pSmad1/5/8 (red). All cells were pretreated with BMP-2 on antifouling TCPS for 1 day. Sample 1 d: pretreated cells were transferred onto adhesive TCPS in the absence of BMP-2 for 1 day. Sample 1 d-1d: pretreated cells were subsequently cultured on antifouling TCPS without BMP-2 for another day before being transferred to adhesive TCPS without BMP-2 for 1 more day. Sample 1 d + BMP-2: positive control, pretreated cells were transferred onto adhesive TCPS in the presence of BMP-2 for 1 day. For nuclear-to-cytoplasmic ratios, n = 10, one-way ANOVA followed by post hoc Tukey's multiple comparisons test.

Immunostaining for osterix

C2C12 cells were initially stimulated with BMP-2 on antifouling TCPS (Anti) and then transferred onto adhesive TCPS (non-treated TCPS) without further BMP-2 stimulation. Osteogenic differentiation was monitored *via* osterix nuclear localization. In line with nuclear accumulation of YAP, only well spread, pretreated cells exhibited high osterix localization (Supplementary Fig. 12).

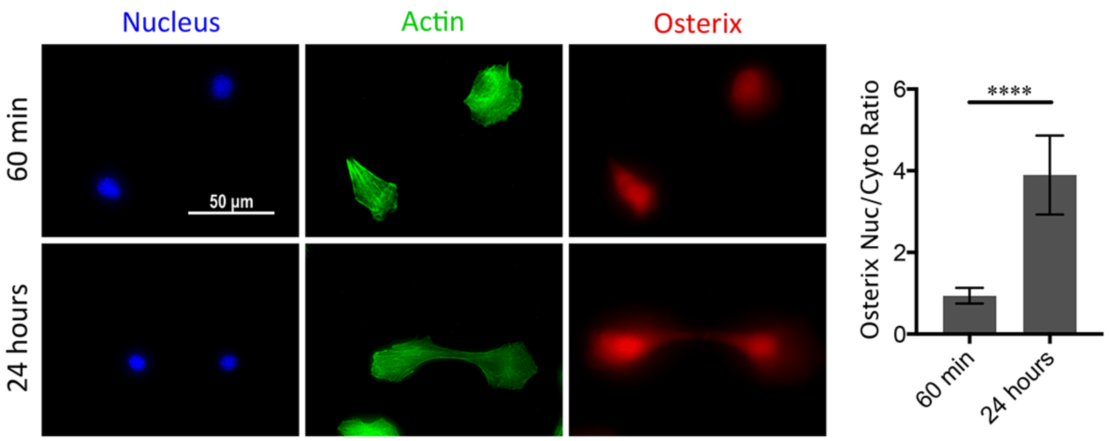

Supplementary Fig. 12 Representative immunofluorescence images of BMP-2 pretreated (1 day) C2C12 cells stained with DAPI (blue), Alexa Fluor 488 Phalloidin (green), and anti-Osterix (red) after being transferred onto adhesive TCPS for 60 min or 24 hours. Corresponding nuclear-to-cytoplasmic ratios of Osterix are graphed, n = 10, Welch's t-test, ****P<0.0001.

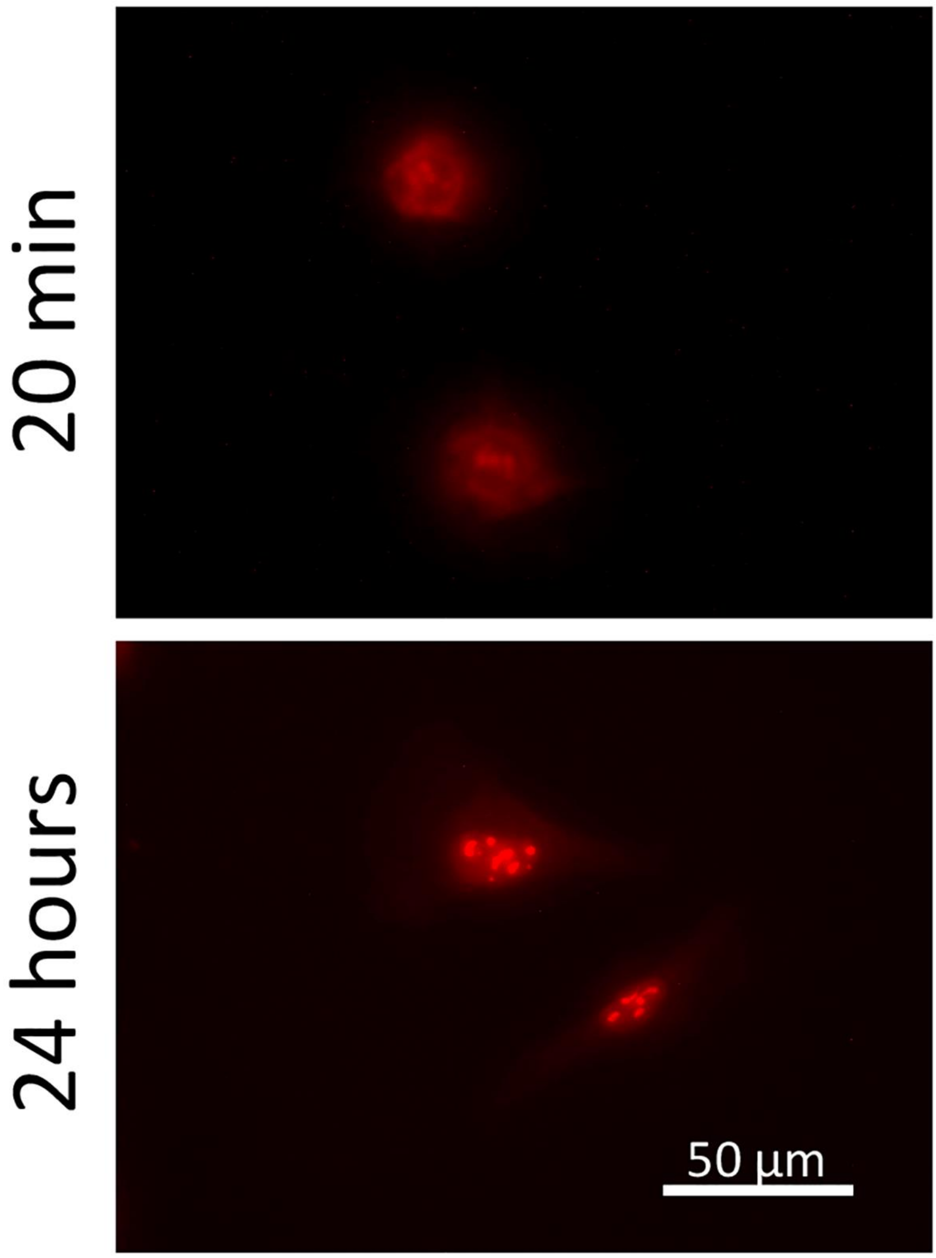

Supplementary Fig. 13 High magnification images of YAP expression in Figure 7e.

Mesenchymal stem cells (MSCs) stimulated by BMP-2

Similar to the experimental setup in section 2.7, human MSCs were initially stimulated with BMP-2 on antifouling TCPS for 1 day and then transferred onto adhesive TCPS without further BMP-2 stimulation. In the lacks of cellular tension, MSCs should tend to differentiate to adipocytes. The BMP-2 treatment decreased the adipogenic differentiation of the cells cultured on antifouling TCPS as indicated by oil Red O assay (Supplementary Fig. 14 and 15). The BMP-2 stimulated and transferred cells showed higher ALP activity than the cells cultured in the same way but without BMP-2 stimulation (Supplementary Fig. 15-17). The MSCs continuously treated with BMP-2 exhibited highest osteogenic and lowest adipogenic differentiation, because the cell proliferation was not inhibited in these experiments. Please note, MSCs grow much slower than C2C12 cells and mitomycin C affected the activity of MSCs. The experiments without mitomycin C treatment are more closed to practical applications. Nevertheless, subsequent BMP-2 and mechanical stimulation can enhance osteogenic differentiation and inhibit adipogenic differentiation of MSCs.

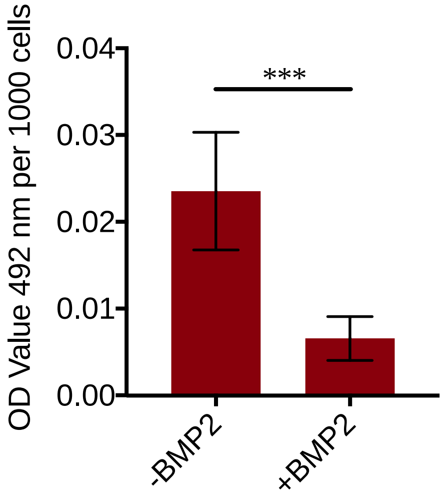

Supplementary Fig. 14 Quantitative assay of oil red O activity (indicating adipogenic differentiation) of MSCs treated with or without BMP-2 for 1 day on antifouling TCPS and were further cultured on antifouling TCPS in growth media for 4 more days. Since the cells cannot adhere, it is impossible to image the Oil Red O stained cells on TCPS (n = 3, 2 technical replicates, Welch's t-test).

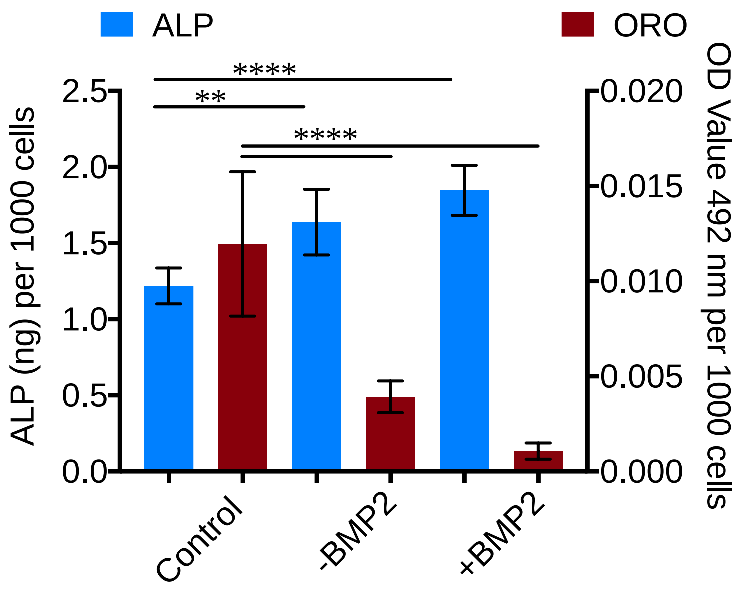

Supplementary Fig. 15 Quantitative assay of oil red O (ORO) and ALP activity of MSCs pretreated with BMP-2 on antifouling TCPS in growth media for 1 day and then transferred onto adhesive TCPS in the presence (+BMP-2) or absence (-BMP-2) of BMP-2 in 1:1 mix ostegenic/adipogenic media for the other 9 days. The cells in the control group were cultured in the same way but without any BMP-2 treatment (n = 2-3, 2 technical replicates, one-way ANOVA followed by post hoc Tukey's multiple comparisons test).

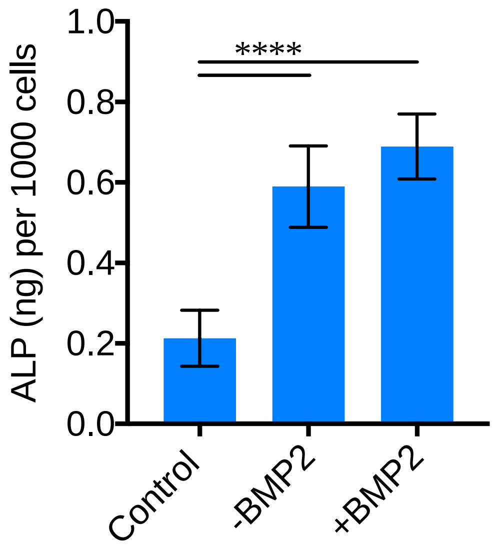

Supplementary Fig. 16 ALP quantitative assay for MSCs pretreated with BMP-2 on antifouling TCPS in growth media for 1 day and then transferred onto adhesive TCPS in the presence (+BMP-2) or absence (-BMP-2) of BMP-2 in growth media for the other 6 days. The cells in the control group were cultured in the same way but without any BMP-2 treatment (n = 2-3, 2 technical replicates, one-way ANOVA followed by post hoc Tukey's multiple comparisons test).

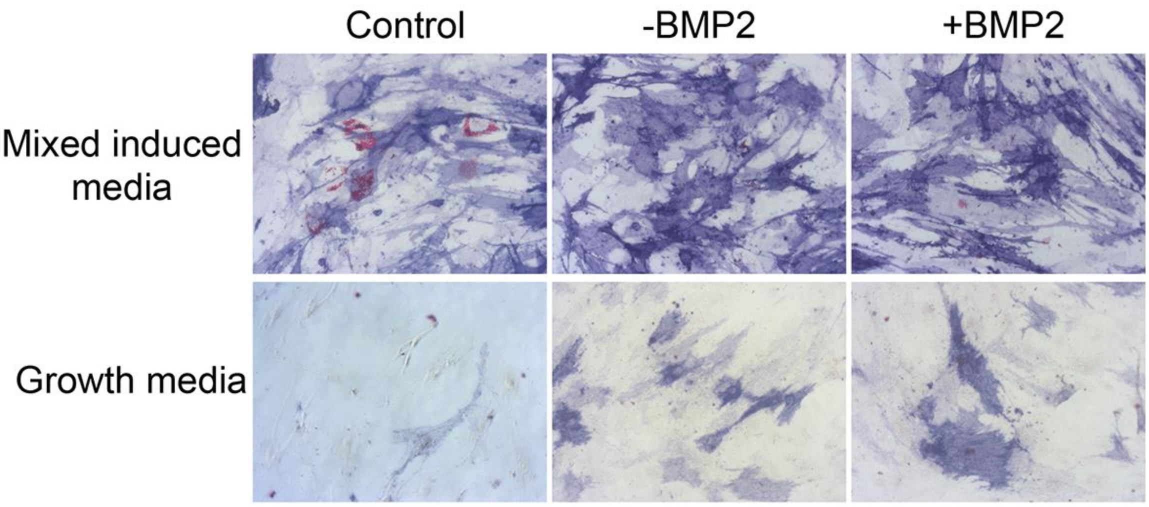

Supplementary Fig. 17 Representative images of ALP and oil red O staining for MSCs pretreated with BMP-2 on antifouling TCPS in growth media for 1 day and then transferred onto adhesive TCPS in the presence (+BMP-2) or absence (-BMP-2) of BMP-2 in 1:1 mix ostegenic/adipogenic media for the other 9 days (upper) or growth media for the other 6 days (lower). The cells in the control group were cultured in the same way but without any BMP-2 treatment.

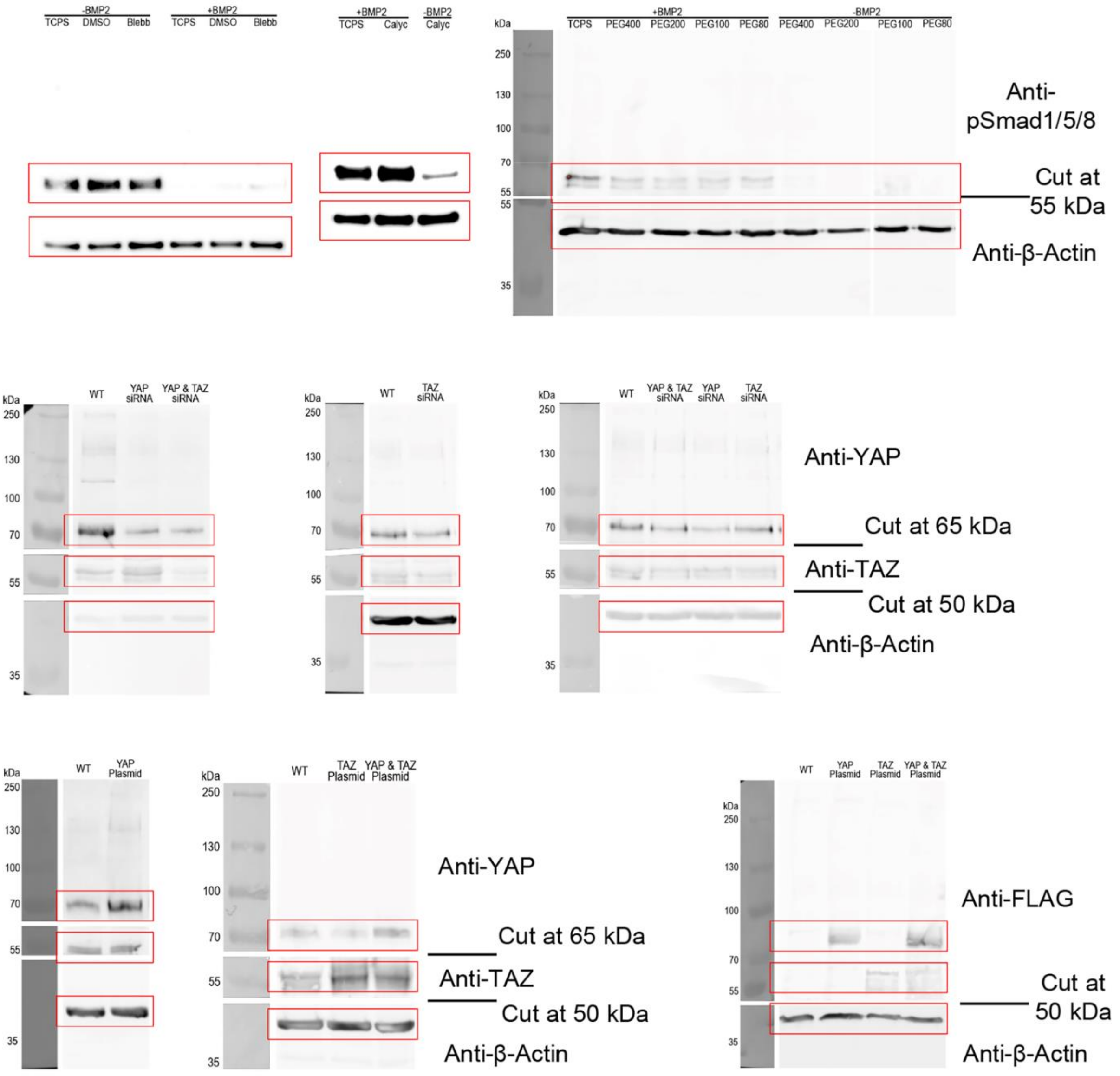

Supplementary Fig. 18 Full scans of uncropped Western blots presented in the main figure. The membranes were cut at the indicated position before staining. The molecular weight markers were imaged as digital photos, after imaging the bands by luminescence.

Supplementary Table 1 Nucleotide sequences of primers for cloning and qRT-PCR.

Gene	Type	Primers (5' to 3')
RUNX2	Forward	CGGCCCTCCCTGAACTCT
	Reverse	TGCCTGCCTGGGATCTGTA
ALP	Forward	CCAACTCTTTTGTGCCAGAGA
	Reverse	GGCTACATTGGTGTTGAGCTTTT
OPN	Forward	ACTCCAATCGTCCCTACAGTCG
	Reverse	TGAGGTCCTCATCTGTGGCAT
GAPDH	Forward	AGGTCGGTGTGAACGGATTTG
	Reverse	TGTAGACCATGTAGTTGAGGTCA

Reference

[1] T. Katagiri, A. Yamaguchi, M. Komaki, E. Abe, N. Takahashi, T. Ikeda, V. Rosen, J. M. Wozney, A. Fujisawasehara, T. Suda, *J. Cell Biol.* **1994**, *127*, 1755.

[2] S. Dupont, L. Morsut, M. Aragona, E. Enzo, S. Giulitti, M. Cordenonsi, F. Zanconato, J. Le Digabel, M. Forcato, S. Bicciato, N. Elvassore, S. Piccolo, *Nature* **2011**, *474*, 179.
